# Spatial‐Compatibility‐Assisted Molecular Intercalation in MXenes

**DOI:** 10.1002/adma.74797

**Published:** 2026-09-04

**Authors:** Minhao Sheng, Xiaoqing Bin, Xiangyang Liu, Yingji Zhao, Wenxiu Que, Yusuke Asakura, Yusuke Yamauchi

**Affiliations:** ^1^ Electronic Materials Research Laboratory Key Laboratory of the Ministry of Education International Center for Dielectric Research Shaanxi Engineering Research Center of Advanced Energy Materials and Devices School of Electronic Science and Engineering Xi'an Jiaotong University Xi'an P. R. China; ^2^ Department of Materials Process Engineering Graduate School of Engineering Nagoya University Nagoya Japan; ^3^ Australian Institute for Bioengineering and Nanotechnology (AIBN) The University of Queensland Brisbane Queensland Australia; ^4^ Department of Chemical and Biomolecular Engineering Yonsei University Seoul South Korea

**Keywords:** crystal structure, energy storage, etching, materials science, molecule, nanotechnology, small molecule, steric effects, taxonomy

## Abstract

MXenes have emerged as versatile platforms for electrochemical energy storage, stimuli–responsive systems, and nanofluidic technologies, owing to their compositionally tunable surfaces and diverse intercalation chemistries. Although ion intercalation in aqueous or organic media is widely employed to modulate the interlayer structure and electronic state of MXenes, the identification of compatible intercalants remains largely empirical. A generally accepted quantitative framework for evaluating the feasibility of guest incorporation into MXene hosts has not yet been established. Here, we propose a general steric–compatibility criterion, in which intercalation becomes geometrically feasible when the effective 3D size of an intercalant molecule, as defined from its molecular crystal structure (i.e., considering a single molecule in the crystalline state), is comparable to or smaller than the interlayer spacing (Δd) created by Al extraction in the parent MAX phase. Guided by this principle, a series of sterically compatible small molecules can be screened, and their intercalation/etching behavior can be rationalized. This study establishes a universal geometry–based guideline for evaluating guest–host compatibility in 2D MXene systems and lays the foundation for predictive design of MXene intercalation chemistries.

## Graphical abstract

This work demonstrates a solvent–free NH_4_F–mediated route for simultaneous Al removal and molecular intercalation in MXenes. Intercalants are selected by comparing their crystallographic X, Y, and Z dimensions with the MXene interlayer spacing, establishing a spatial‐compatibility criterion that rationalizes guest–host matching and enables predictive synthesis of expanded, readily delaminated MXene nanosheets.

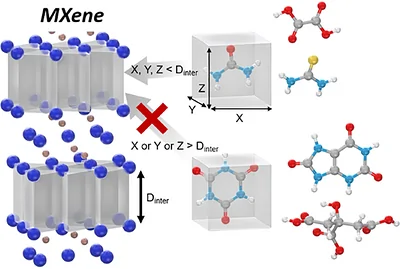

## 1 Introduction

MXenes constitute a broad family of 2D transition–metal carbides, carbonitrides, and nitrides obtained by selectively etching the A–site layers from their parent MAX phases. Owing to their metallic conductivity, tunable surface terminations, and mechanically robust laminated architecture, MXenes have demonstrated exceptional performance in electrochemical energy storage [1, 2, 3], electromagnetic interference (EMI) shielding [4, 5] and absorption [6, 7], heterogeneous catalysis [8], gas sensing [9], seawater desalination [10], water purification [11, 12], and emerging biomedical applications [13]. Since the first report [14] of accordion–like Ti_3_C_2_T*_x_* MXene generated by HF etching in 2011, diverse chemical etching strategies [15, 16] have been developed to access a much wider range of MXene compositions. The layered galleries formed by selective removal of Al atoms enable the intercalation of a large variety of guest species, including metal cations [17, 18, 19] and organic molecules [20, 21, 22], thereby producing highly expanded few–layer structures. Typically, Shahzad et al. introduced the minimally intensive layer delamination (MILD) protocol, in which Li^+^ ions intercalate into Ti_3_C_2_T*_x_* to expand the interlayer spacing and facilitate delamination [23], establishing this approach as the dominant route for producing high–quality monolayer and few–layer MXenes. Beyond Li^+^, macromolecular organic bases [21], ionic liquids [24], amines [20], organic polar solvents [25, 26], and carbonate salts [27] have been employed as post–treatment intercalants. More recently, low–temperature molten–salt etching has been achieved using NH_4_HF_2_ [28] and NH_4_HF_2_–NH_4_F [29] eutectic media. However, these approaches primarily address the efficiency of MAX–phase etching, while subsequent intercalation and delamination are still required to obtain few–layer MXenes. Although various species, ranging from small water molecules to bulky organic molecules, have been demonstrated to enter MXene interlayer galleries, the selection of suitable intercalants remains largely empirical. Moreover, a quantitative and predictive guideline correlating molecular size or geometry with the interlayer structural parameters of MXenes has not yet been established.

MXene intercalation processes in aqueous or organic media often suffer from sluggish kinetics and solvent‐induced limitations [30, 31]. In this Article, we introduce a solvent–free one–step etching/intercalation approach to produce readily exfoliable multilayer MXenes, wherein ammonium fluoride acts simultaneously as an etchant and as a carrier for small–molecule guest intercalants, enabling concurrent Al removal and mild exfoliation of diverse MAX phases. We propose a general steric–compatibility criterion, in which intercalation becomes sterically feasible when the effective 3D size of an intercalant molecule, as defined from its molecular crystal structure (i.e., considering a single molecule in the crystalline state), is comparable to or smaller than the interlayer spacing (Δd) created by Al extraction in the parent MAX phase. Guided by this principle, a series of small molecules are demonstrated to intercalate into MXene interlayers. Using this approach, a broad family of multilayer MXenes including Ti_3_C_2_T*_x_*, Mo_2_TiC_2_T*_x_*, V_2_CT*_x_*, V_4_C_3_T*_x_*, Nb_2_CT*_x_*, and Nb_4_C_3_T*_x_* is successfully synthesized. Moreover, the as–obtained multilayer Ti_3_C_2_T*_x_* can be readily delaminated into few–layer nanosheets through simple ultrasonication. This work proposes a geometry‐based empirical descriptor to rationalize the compatibility between guest molecules and 2D MXene hosts.

## 2 Results and Discussion

Traditional synthesis of MXenes is typically carried out in liquid media, where the A–site elements of MAX phases are selectively etched. However, under such solvent–based conditions, the coexistence of metal cations and solvent molecules makes the intercalation behavior of MXenes highly complex [32]. For instance, in aqueous systems, both the molecular configuration of water and the solvation structure of hydrated cations must be taken into account when considering interlayer structural evolution [33, 34]. The effective size of hydrated cations is particularly difficult to define, as their solvation shells are highly dynamic, environment–dependent, and can undergo partial dehydration or complex interactions with MXene surface terminations during intercalation [35, 36, 37]. To elucidate the intercalation mechanism on MXenes, we employed a solvent–free low‐temperature molten salt etching strategy. Unlike conventional solvent–based routes, this approach eliminates interference from solvent molecules, thereby providing a chemically simplified platform for probing the fundamental relationship between the MXene interlayer structural evolution and the physicochemical characteristics of the intercalated molecules or ions.

### 2.1 Thermodynamic Prediction of Molten Salt Etching via NH_4_X (X = F, Cl, Br, I)

Ammonium halides (NH_4_X), owing to their low melting points and favorable electron–donating characteristics, partially dissociate into halide and ammonium ions at low temperatures [38]. This dissociation provides a reactive environment capable of extracting the A–site elements (Al) from MAX phases and initiating MXene formation. In MAX crystal structures such as Ti_3_AlC_2_, Mo_2_TiAlC_2_, V_2_AlC, V_4_AlC_3_, Nb_2_AlC, and Nb_4_AlC_3_ (Figures 1a1 and S1–S6.), Al atoms occupy trigonal–prismatic sites coordinated by six transition metal atoms rather than existing in a free metallic state. Halide anions (X^−^) must overcome substantial steric and electrostatic barriers to diffuse through the narrow M–Al lattice gaps and access the Al sites [39]. In Ti_3_AlC_2_, the Ti–Al spacing (D_Ti‐Al_) is roughly 2.90 Å (Figures 1a1 and S1), while the ionic diameters of F^−^, Cl^−^, Br^−^, and I^−^ are approximately 2.72 Å, 3.62 Å, 3.90 Å, and 4.32 Å, respectively. Only F^−^ possesses a steric diameter smaller than the Ti–Al bond length, enabling it to reach and coordinate with the Al center (Figure 1a2) and thereby promote Ti–Al bond cleavage. Similarly, the corresponding M–Al spatial distances (D_M‐Al_) were calculated based on the crystal structures of the other MAX phases. Accordingly, Mo_2_TiAlC_2_, V_2_AlC, V_4_AlC_3_, Nb_2_AlC, and Nb_4_AlC_3_ are likewise predicted to be etchable in NH_4_F, as the F^−^ ionic diameter is smaller than the corresponding D_M–Al_ values (Figures S2–S6). Thermodynamic calculations performed using HSC 6.0 (Tables S1–S4 and Figure 1b) provide the Gibbs free energy changes (ΔG_r_) associated with the etching of Al by NH_4_X, as represented in Equation (1) (Note: all AlX_3_ species are assumed to be in the gaseous state at 200°C). Although Al does not exist as a free metal in MAX phases, the ΔG_r_ values nevertheless offer first–order insight into the thermodynamic feasibility of the reaction [40]. Among the ammonium halides, NH_4_F exhibits a markedly more negative ΔG_r_ (ΔG_r_<0, T = 0–200°C) than NH_4_Cl, NH_4_Br, and NH_4_I, indicating that fluoride species promote a strongly exergonic and thermodynamically favorable etching process. Higher temperatures further improve etching efficiency in practical experiments. From a purely thermodynamic standpoint, all NH_4_X salts are capable of removing Al layers at approximately 200°C.

(1) $$\mathrm{Al}+3{\mathrm{NH}}_{4}X\to \mathrm{Al}X_{3}\left(g\right)+1.5H_{2}\left(g\right)+3{\mathrm{NH}}_{3}\left(g\right)$$

**FIGURE 1:**
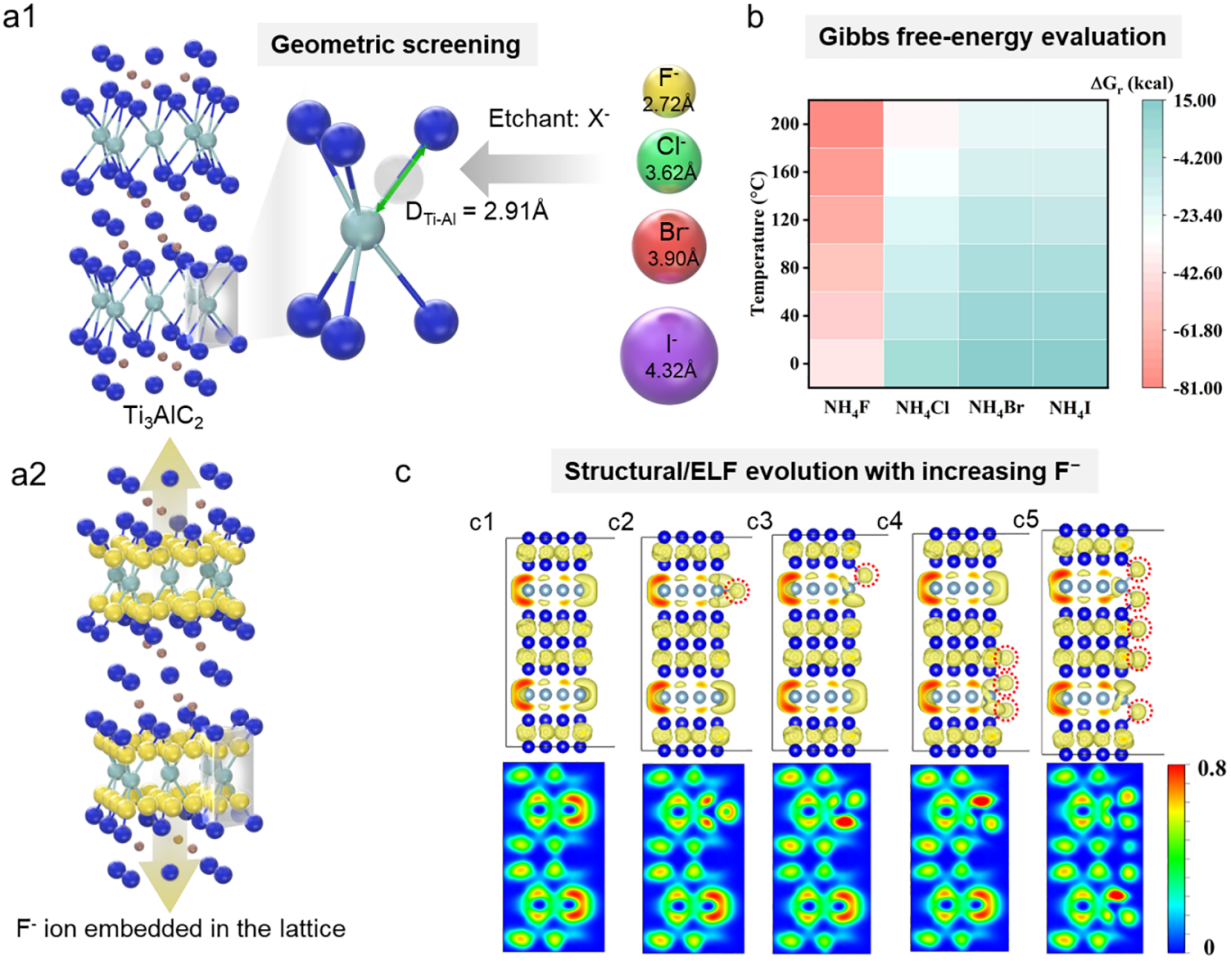
Prediction of the possibility of Al etching by ammonium halides based on thermodynamic Gibbs free energy and the mechanism of Al etching by fluoride ions studied via DFT calculations. Ti atom: deep blue; Al atom: sky blue; C atom: brown; F atom: yellow. (a1–2), the occupancy position of Al in the Ti_3_AlC_2_ crystal structure and comparison between the theoretically calculated Ti–Al bond length and the ionic diameters of halide ions, indicating that the F^−^ ion can most readily access the Ti–Al region and coordinate with Al sites. (b) The Gibbs free energy profiles (0°C–200°C) for the reaction between NH_4_X and Al obtained from HSC Chemistry calculations. (c) Lowest‐energy atomic configurations and their corresponding ELF maps of pristine Ti_3_AlC_2_ and etched Ti_3_AlC_2_ with increasing amounts of F^−^ions. Structures containing (**c1**) 0, (**c2**) 1, (**c3**) 2, (**c4**) 3, and (**c5**) 11 F^−^ ions per unit cell. Red circles represent fluorine atoms.

To further elucidate the etching mechanism, density functional theory (DFT) calculations were performed for the interaction between Ti_3_AlC_2_ and F^−^ in NH_4_F. Electron localization function (ELF) maps (Figure 1c) reveal the bonding interactions between MAX phases and F^−^ ions during the etching process, where ELF = 1 corresponds to complete localization, ELF = 0.5 represents homogeneous electron gas, and ELF = 0 refers to complete delocalization [41, 42]. In pristine Ti_3_AlC_2_ MAX (Figure 1c1), strong Ti–C covalent bonds (ELF 0.68–0.72) contrast sharply with the weaker Ti–Al bonds (ELF 0.48–0.52). Notably, the Ti–Al edge bonds exhibit even lower electron localization (ELF ≈ 0.32), suggesting reduced electron sharing and enhanced metallic character, which may facilitate preferential interaction with nucleophilic F^−^ ions during the etching process. Upon progressive introduction of F^−^ (Figure 1c2–1c5), strong Al–F bonds form, which disrupt the original Ti–Al bonds. This preferential interaction facilitates the selective Ti–Al bond cleavage and promotes the removal of Al layers during the etching process.

### 2.2 Solvent‐free NH_4_F Etching for Ti_3_C_2_T*_x_* MXene Synthesis

Ti_3_AlC_2_ was selected as a representative MAX precursor to demonstrate solvent–free molten salt etching for MXene synthesis. Guided by the thermodynamic feasibility calculations, we employed a sealed autoclave system to etch Ti_3_AlC_2_ under solvent‐free conditions with ammonium halide salts. Prior to etching, high–quality MAX phases were annealed via high–temperature solid–state reaction. The detailed preparation procedures are shown in the Experimental Section. Ti_3_AlC_2_ and NH_4_X powders were loaded into a Teflon–lined autoclave and shaken to ensure homogeneity. Given the strong temperature dependence of the etching process, the reaction temperature was fixed at 200°C. The morphology of Ti_3_AlC_2_ and the corresponding etching products were examined by scanning electron microscopy (SEM). Pristine Ti_3_AlC_2_ exhibits a densely stacked morphology (Figure S7), whereas NH_4_F–etched samples display characteristic accordion–like layers indicative of MXene formation (Figure S8). Elemental mappings of individual Ti_3_AlC_2_ platelets show uniform distribution of Ti, C, and Al (Figure S9), whereas NH_4_F–etched samples exhibit heterogeneous Al distribution (Figure S10). Moreover, the Ti/Al atomic ratio increases significantly compared to the pristine Ti_3_AlC_2_, confirming substantial Al extraction. The slight residual Al content and its heterogeneous distribution indicate that a fraction of the particles is not fully reacted. In contrast, the samples treated with NH_4_Cl, NH_4_Br, and NH_4_I retain the characteristic bulky MAX–phase morphology, as observed by SEM images (Figures S11–S13). These samples show uniform Al distribution (Figures S14–16) and exhibit significantly lower Ti/Al ratios than those produced using NH_4_F (Figure S17). X–ray diffraction (XRD) patterns (Figure S18) confirm that the as–annealed Ti_3_AlC_2_ possesses high crystallinity. The prominent reflections at 9.50°, 19.08°, 33.92°, 36.66°, 38.89°, 41.62°, 48.37°, and 56.27° correspond to the (002), (004), (101), (103), (104), (105), (107), and (109) planes, respectively. After NH_4_F etching, the MAX–phase reflections decrease markedly in intensity, showing the most pronounced attenuation among all etching treatments. The peaks originating from the phase formed after Al etching are extremely weak in intensity, implying a random or loose stacking of Ti_3_C_2_T*_x_* layers. These experimental observations corroborate the theoretical predictions. F^−^ ions can penetrate the Ti–Al interlayer owing to their small ionic diameter, thereby facilitating the extraction of Al atoms.

### 2.3 Evaluation of Molecule Compatibility With MXene Spacing

Molten–salt etching with NH_4_F does not produce the substantial interlayer expansion or the subsequent exfoliation into few–layer structures commonly observed in traditional liquid–phase etching systems, such as HCl/LiF [18]. Considering that expansion of the interlayer spacing is the most energy–demanding step during intercalation, intercalation is highly dependent on the interlayer spacing of multilayered MXenes [27, 43]. The interlayer gallery (D_inter_) of Ti_3_C_2_T*_x_* MXene can be used as a geometric descriptor for screening intercalants with molecular diameters not exceeding 4.60 Å, as estimated from the interlayer spacing of Ti_3_C_2_T*_x_* MXene after Al removal (Figure 2). Here, we do not subtract a Ti “atomic size” because atomic radii (covalent, van der Waals, or ionic) are model–dependent and do not define a rigid boundary in solids. Instead, we use the center–to–center separation as a geometric length–scale descriptor to enable consistent comparisons across systems. This estimated value is derived from the original Ti_3_AlC_2_ MAX crystal structure (Figure S1), because no expansion of the MXene interlayer spacing was observed after etching with ammonium fluoride alone. In this simplified D_inter_ descriptor, surface terminations are not separately parameterized. Instead, their baseline contribution is accounted for by using the NH_4_F–only etched MXene as the reference state, for which no obvious shift of (002) peak was observed (Figure S18). As discussed above, NH_4_F treatment results in loosely stacked Ti_3_C_2_T*_x_* MXene layers, a structural state that is advantageous for subsequent intercalation of other species to further expand the interlayer spacing. In selecting potential intercalants, it is important to note that, unlike metal cations (Li^+^, Na^+^, and K^+^), which can intercalate readily in aqueous solution, conventional inorganic salts such as NaCl, LiCl, and KCl are unlikely to insert into MXene interlayers in low–temperature molten salts at 200°C. This limitation arises from the strong Coulombic interactions between cations and anions in ionic crystals, due to high lattice energies and rigid crystalline frameworks. Such strong electrostatic coupling makes it energetically unfavorable for individual ions to dissociate and diffuse into the confined interlayer galleries of MXene. In contrast, molecular crystals, which are stabilized primarily by weak van der Waals forces or hydrogen bonds, can be readily disrupted under mild thermal conditions [44]. Therefore, evaluating whether molecular crystals can intercalate under low–temperature and anhydrous conditions represents a fundamentally different strategy from traditional liquid–phase exfoliation. Smaller–sized intercalants typically experience less steric hindrance and can access a wider range of interstitial and interlayer sites [43, 45].

**FIGURE 2:**
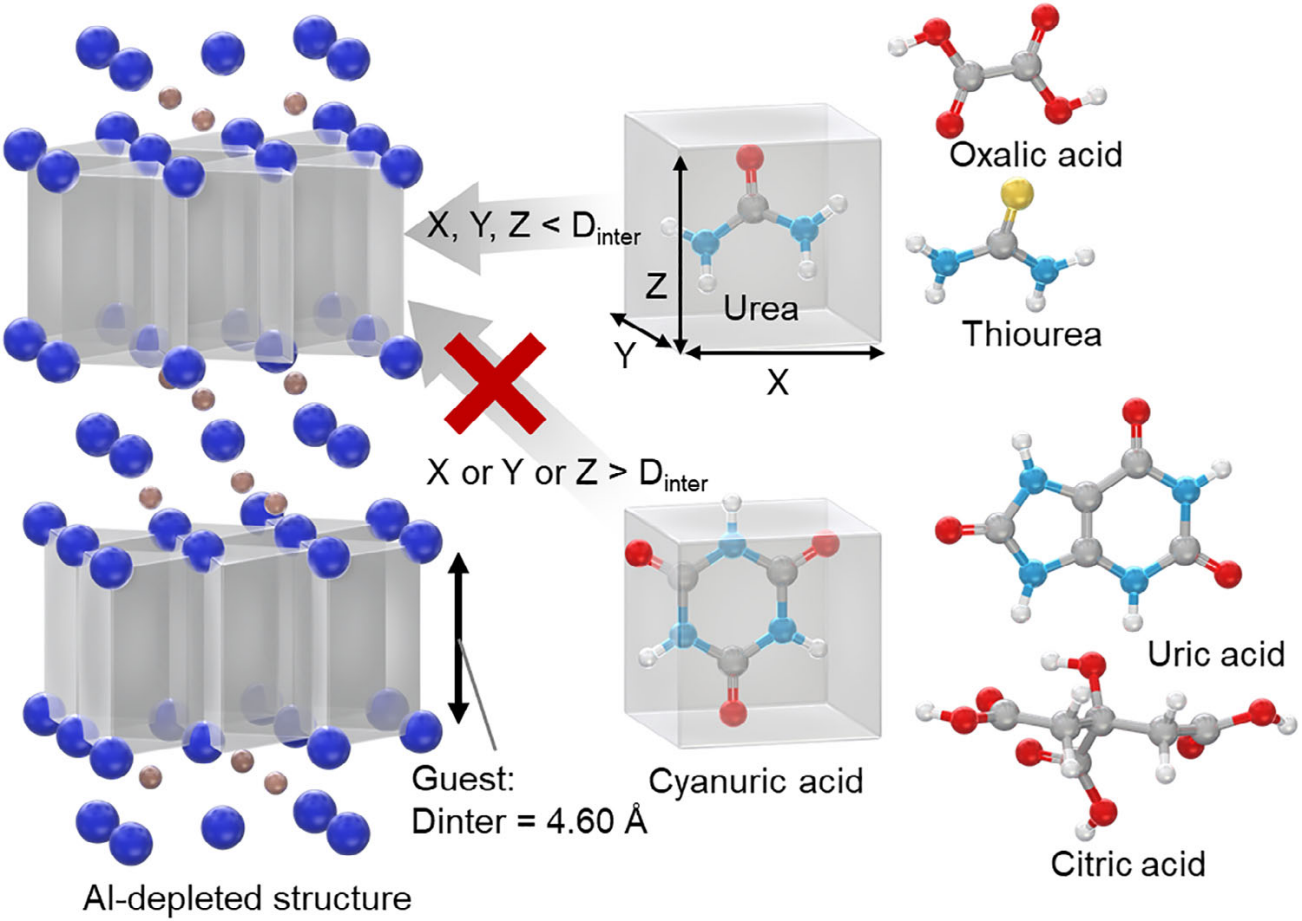
Screening and size–quantitative evaluation criteria for MXene intercalant molecules. Ti_3_C_2_T*_x_* MXene crystal structure and the calculated D_inter_ interlayer distance. The evaluation criteria for intercalant guests entering the interlayers of Ti_3_C_2_T*_x_* MXene. Intercalation feasibility is assessed by comparing the molecular dimensions (X, Y, Z) of candidate intercalants with D_inter_.

In this work, we propose a general steric–compatibility criterion, in which intercalation becomes thermodynamically accessible when the effective 3D size of an intercalant molecule is comparable to or smaller than the interlayer spacing (D_inter_ = 4.6 Å (Figure 2)) created by Al extraction in the parent MAX phase. Herein, the effective 3D molecular size is estimated as the occupied spatial dimensions (X, Y, and Z) of a single molecule, derived from crystallographic data of its molecular crystal structure (Figures S19–S25). For simplicity, we do not consider atomic radii, and instead perform the calculations solely based on the Cartesian (fractional) coordinates provided in the CIF file. It should be noted that the molecular dimensions extracted from solid–state crystal structures are used here only as approximate descriptors of the intrinsic size and shape of the initial additives. Under molten–salt etching conditions at 200°C, the additives are not fixed in their crystallographic conformations, but may exist as flexible conformers or rotamers in the molten medium. Once the molecular crystals reach the molten state, their long–range crystallographic order is lost, and continuous translational, rotational, conformational, and rearrangement motions may occur. In addition, under the sealed reaction conditions, the additives and their molecule–derived species may exist in a dynamic equilibrium between molten and vapor phases. Therefore, the present molecular–size analysis should be regarded as a qualitative or semi–quantitative descriptor for comparing different additives, rather than as a quantitative prediction of their exact effective dimensions under the reaction conditions. This provides a straightforward and reproducible metric, although the actual accessible size may depend on the chosen surface definition and molecular conformation. Several representative molecules were selected as candidate intercalants (Figures 2 and S19–25), including small molecules (urea, thiourea, and oxalic acid), medium–sized molecules (uric acid, cyanuric acid, and citric acid), and large molecules (cetyltrimethylammonium bromide). According to the molecular size, only the small molecules (urea, thiourea, and oxalic acid) can meet the intercalation criterion (X, Y, Z < D_inter_). Furthermore, we calculated the interlayer gallery spacing D_inter_ for several MXenes (Mo_2_TiC_2_T*_x_*: 4.35 Å, V_2_CT*_x_*: 4.31 Å, V_4_C_3_T*_x_*: 4.36 Å, Nb_2_CT*_x_*: 4.47 Å, and Nb_4_C_3_T*_x_*: 4.52 Å) based on their crystal structures (Figures S1–S6). Analogously, only the small molecules are predicted to intercalate into these MXene hosts.

### 2.4 Intercalation of Ti_3_C_2_T*_x_* MXene With Compatible Molecules Through NH_4_F Etching

To evaluate the reliability and applicability of the intercalation benchmark, we subsequently carried out the corresponding experimental validations. Building on the aforementioned etching of MAX phases with ammonium fluoride, we concurrently implemented NH_4_F etching–assisted intercalation. Figure 3a presents the XRD patterns of Ti_3_AlC_2_ MAX after reaction with NH_4_F and small intercalant molecules (urea and thiourea) at 200°C for 2 h. Compared with the pristine MAX, most diffraction peaks of NH_4_F+urea and NH_4_F+thiourea disappear in the final product, leaving only the 002 reflection and a few broad and low–intensity peaks in the 2θ range from 5° to 60°. The XRD patterns of Ti_3_C_2_T*_x_* MXene (NH_4_F+urea and NH_4_F+thiourea) show a noticeable shift of the (002) peak toward lower 2θ values compared with Ti_3_AlC_2_, indicating an increased interlayer spacing due to the removal of Al layers and the subsequent intercalation of molten small molecules during the synthesis process. According to Bragg's law (*λ* = 1.5406 Å), the (002) lattice plane spacing of Ti_3_C_2_T*_x_* MXene increases from 9.28 Å in the Ti_3_AlC_2_ precursor to 13.11 Å (NH_4_F+urea) and 12.94 Å (NH_4_F+thiourea). Compared with NH_4_F alone, small–molecule intercalation significantly enhances the etching rate of MAX phases, primarily because low–melting molecular crystals can codiffuse with NH_4_F through the MXene galleries. SEM images of the etching products and the corresponding EDS elemental mappings are shown in Figures 3b, S26, and S27. After etching in NH_4_F+urea and NH_4_F+thiourea, the Ti_3_AlC_2_ sample transforms into an accordionlike microstructure, similar to the MXenes obtained by etching in F–containing corrosive liquid etchants [3]. Furthermore, the significant decrease in the Al atomic ratio (Figures S26–3 and S27–3) further supports the high efficiency of etching promoted by small‐molecule intercalation. The multilayer flake microstructure of the Ti_3_C_2_T*_x_* MXene obtained from NH_4_F+thiourea etching is clearly observed in transmission electron microscopy (TEM) images, as shown in Figure 3c.

**FIGURE 3:**
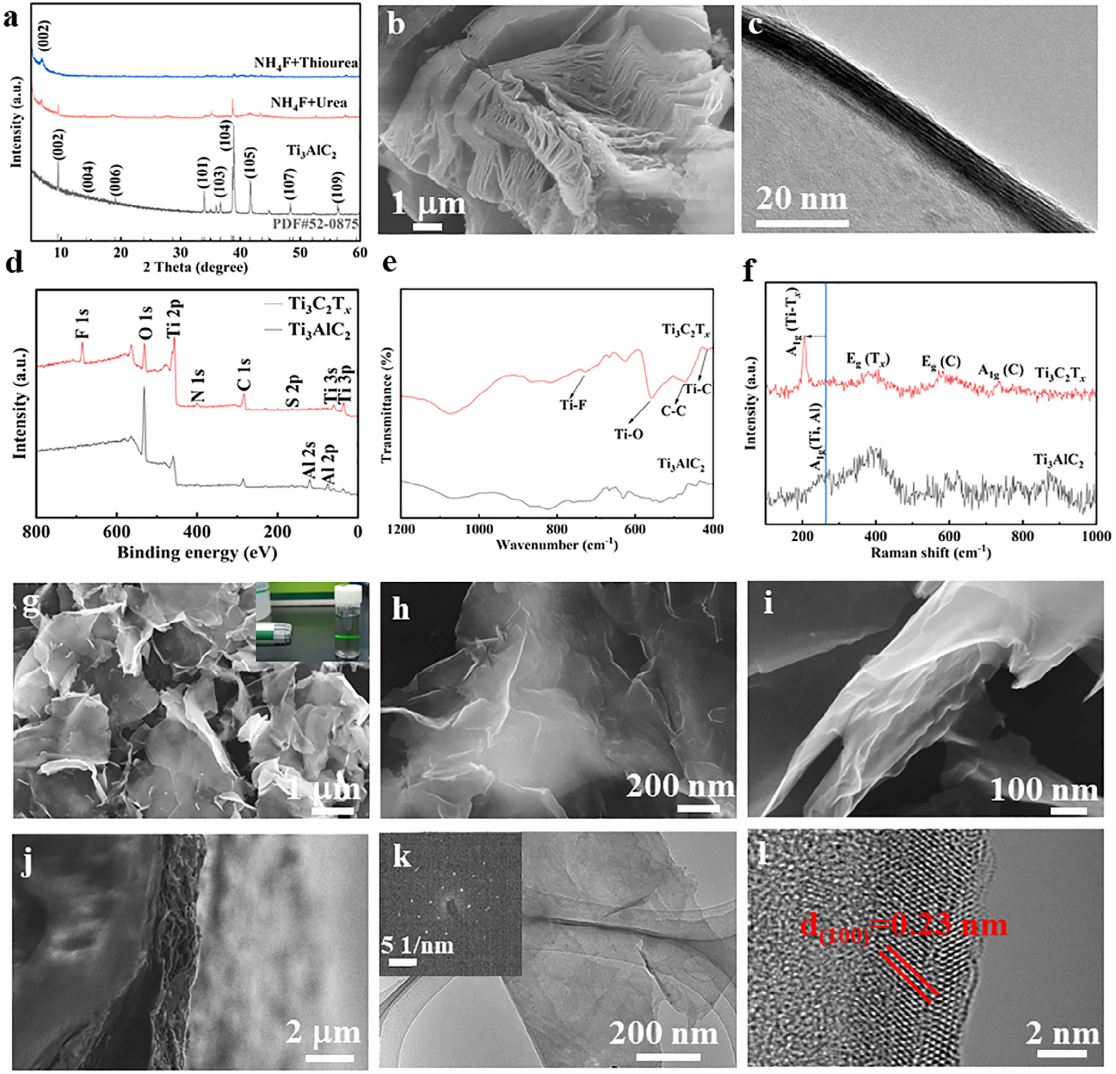
Experimental verification and characterization of small–molecule intercalation into Ti_3_C_2_T*_x_* MXene under low–temperature molten–salt conditions. (a) XRD patterns of the original Ti_3_AlC_2_ MAX phase and the Ti_3_C_2_T*_x_* MXenes etched using NH_4_F + urea and NH_4_F + thiourea. (b) SEM image of Ti_3_C_2_T*_x_* MXene etched using NH_4_F + thiourea. (c) TEM image of Ti_3_C_2_T*_x_* MXene multilayer nanosheet etched using NH_4_F + thiourea. (d) XPS survey, (e) FTIR, and (f) Raman spectra of Ti_3_AlC_2_ MAX and Ti_3_C_2_T*_x_* MXene etched using NH_4_F + thiourea. (g–i) The SEM images of exfoliated few–layer Ti_3_C_2_T*_x_* MXene obtained by ultrasonic treatment. The inset shows that the solution exhibits a pronounced Tyndall effect. (j) The cross‐sectional SEM image of few–layer Ti_3_C_2_T*_x_* MXene. (k) TEM image of the exfoliated Ti_3_C_2_T*_x_* MXene and corresponding SAED pattern. (l) HRTEM image of the exfoliated Ti_3_C_2_T*_x_* MXene.

Thiourea–assisted etching is more efficient than urea–based etching, as evidenced by the XRD patterns (Figure 3a). To further characterize the surface chemistry, the Ti_3_C_2_T*_x_* MXene obtained from NH_4_F+thiourea etching was analyzed by X–ray photoelectron spectroscopy (XPS). For both pristine Ti_3_AlC_2_ and Ti_3_C_2_T*_x_* MXene samples (Figure 3d), Ti 2p, C 1s, and O 1s signals were detected. The disappearance of the Al 2p and 2s signals in Ti_3_C_2_T*_x_* MXene confirms the effectiveness of the low–temperature molten–salt etching, while the appearance of O 1s and F 1s signals is attributed to the formation of surface T*_x_* (–F and –O) terminations. Consistent with prior reports, the Ti 2p spectra (Figure S28a) are deconvoluted into components corresponding to C–Ti–T*_x_*, C–Ti^2+^, C–Ti^3+^, and TiO_2_. The corresponding fitting parameters are summarized in Table S5. In addition, the O 1s (Figure S28d) and C 1s (Figure S28b) spectra show the presence of Ti–O surface terminations in the Ti_3_C_2_T*_x_* sample, while the F 1s spectra (Figure S28e) reveal the presence of surface –F terminations. For the N (Figure S28c) and S (Figure S28f) heteroatoms, trace amounts of Ti–N and C═S groups are detected on the MXene surface, likely originating from thiourea–derived species during intercalation [34, 46, 47]. Analysis of the XPS–derived elemental composition (Table S5) yields a surface composition of MXene is determined to be Ti_3_C_2_O_0.57_OH_0.62_F_1.04_N_0.08_S_0.12_.

Compared with the FTIR spectrum of Ti_3_AlC_2_ (Figure 3e), the emergence of Ti–F (728 cm^−1^) and Ti–O (560 cm^−1^) bands in Ti_3_C_2_T*_x_* MXene confirms the successful removal of Al layers and incorporation of surface terminations during the etching process [48]. Moreover, the Raman spectrum of Ti_3_C_2_T*_x_* MXene (Figure 3f) differs markedly from that of the precursor MAX phase Ti_3_AlC_2_. The Raman spectrum of Ti_3_C_2_T*_x_* MXene exhibits distinct peaks at ∼206 cm^−1^ (A_1g_, Ti–T*_x_* vibration), ∼382 cm^−1^ (E_g_, T*_x_* termination), ∼580 cm^−1^ (E_g_, C vibration), and ∼734 cm^−1^ (A_1g_, C vibration). The A_1g_(Ti, Al) mode corresponding to the ω_4_ vibration of the MAX phase vanishes and is replaced by a sharper Ti–T*_x_* vibration [49]. Collectively, these results confirm that the proposed low–temperature molten salt etching process efficiently removes Al layers from Ti_3_AlC_2_, resulting in Ti_3_C_2_T*_x_* MXene enriched with surface functional groups.

In contrast to conventional water–free Lewis–acid molten–salt routes, which often require additional post‐intercalation/exfoliation steps [40, 50], the NH_4_F–thiourea strategy enables the direct production of few–layer MXene through simple ultrasonic treatment after cleaning the MXene, without relying on additional harmful and expensive organic macromolecular intercalants for post–intercalation and exfoliation [30]. The initially produced accordion–shaped MXene is further exfoliated into thin sheets (Figure 3g–i), and the obvious Tyndall effect confirms its colloidal nature, as shown in the inset of Figure 3g. The exfoliated MXene can also be assembled into a freestanding membrane via vacuum filtration (Figure 3j), although it appears more fragile compared to the membranes formed using the HCl/LiF etching process [51]. TEM image (Figure 3k) reveals transparent Ti_3_C_2_T*_x_* sheets with distinct and clean edges. The selected area electron diffraction pattern (SAED) exhibits sharp reflections corresponding to a hexagonal crystal structure. The high–resolution TEM (HRTEM) image of Ti_3_C_2_T_x_ MXene (Figure 3l) demonstrates lattice fringes with a spacing of 0.23 nm, corresponding to the (100) plane of MXene. These characteristics confirm that small molecules act as intercalants during NH_4_F etching, thereby enabling an efficient pathway for few–layer MXene.

Judging from the abovementioned results, urea and thiourea are believed to act as effective expansion agents during etching, promoting MXene delamination in a manner analogous to hydrated–cation intercalation in aqueous etchants. However, neither obvious MAX nor MXene diffraction peaks are detected in the products obtained from the NH_4_F+oxalic acid system (Figure S29). Instead, only numerous insoluble white NH_4_AlF_4_ aggregates remain after NH_4_F+oxalic acid etching (Figure S30), suggesting complete structural destruction of the MXene. The corresponding elemental mappings (Figure S31) further confirm excessive etching of Ti, as only Al signals remain in the solid residue, providing strong evidence of over–etching behavior. This observation does not contradict our prediction regarding small–molecule intercalation. Rather, the combined interaction between oxalic acid and NH_4_F significantly accelerates the etching process, thereby leading to further attack on Ti species. The different etching rates observed for urea, thiourea, and oxalic acid, under identical NH_4_F–based conditions, likely arise from their intrinsic physicochemical properties, such as melting points and hydrogen‐bonding interactions [34, 46, 47, 52, 53]. It should be noted that the small–molecule additives may not remain completely intact during the molten–salt etching process. At the reaction temperature of 200°C, partial decomposition, protonation, condensation, or other chemical transformations may occur, generating molecule‐derived species that could further modify the local etching environment and facilitate the transport of reactive fluoride–containing species.

Based on the estimated 3D molecular sizes (X, Y, and Z) derived from their molecular crystal structures, we selected three additional small molecules: acetamide (X = 2.65 Å, Y = 1.15 Å, and Z = 1.77 Å), propionamide (X = 3.25 Å, Y = 3.94 Å, and Z = 4.14 Å), and glycine (X = 3.21 Å, Y = 2.74 Å, and Z = 3.71 Å). The estimated dimensions fall within the sterically accessible interlayer space (D_inter_ = 4.6 Å). The specific lattice parameters and 3D spatial information of these individual molecules are provided in Figures S32–S34. Similar to oxalic acid, Ti_3_AlC_2_ rapidly dissolves when reacted with NH_4_F in the presence of these sterically compatible molecules due to severe over‐etching (Figures S35–S38), leaving only aluminum fluoride species such as (N_2_H_5_)_2_AlF_5_, (NH_4_)_3_AlF_6_, and AlF_3_. Additional experiments (Figure S39) further confirmed that the extent of over–etching is strongly dependent on the reaction temperature and time. Prolonging the urea–assisted etching at 200°C to 6 h resulted in severe over–etching, as evidenced by the formation of Al_2_O_3_ and a decrease in the product yield to approximately 10%. In contrast, Ti_3_C_2_T*_x_* formation was observed in the oxalic acid–containing system after treatment at 150°C for 0.5 h, as well as in the acetamide–and propionamide–containing systems after etching at 200°C for 0.5 h, as indicated by the shifted MXene (002) reflections. These results demonstrate that over–etching can be effectively alleviated by lowering the reaction temperature or shortening the etching time. As a control within the same homologous amide series, we further examined butyramide (Figure S40‐1) as a homologous control molecule because it belongs to the same homologous amide series as acetamide and propionamide, possesses the same amide functional group and similar chemical properties, but has larger molecular dimensions (X = 4.61 Å, Y = 5.28 Å, Z = 3.96 Å). Under identical reaction conditions (200°C for 2 h), the butyramide–treated sample (Figure S40‐2) showed neither a discernible shift of the (002) reflection nor appreciable over–etching. Because all three amides were heated above their respective melting points under the reaction conditions, their different behaviors cannot simply be attributed to differences in melting point. Instead, these differences are more likely associated with their molecular dimensions and spatial compatibility with the MXene interlayer galleries. These results further support the existence of a relative size–compatibility trend within a homologous molecular series. To further evaluate the specificity of the small–molecule intercalation strategy, we extend this method to additional molecule systems, including uric acid, cyanuric acid, citric acid, and cetyltrimethylammonium bromide. No shifted MXene (002) diffraction peak (Figure S41‐1) is detected in medium– and large–molecule systems with dimensions exceeding 4.6 Å along any spatial axis, although multilayer and accordion–like MXene is observed. In addition, these systems pose challenges in removing the poorly soluble intercalant molecules and the decomposition by–products they generate (Figure S41‐2).

### 2.5 Synthesis of Diverse MXenes (Mo_2_TiC_2_T*_x_*, V_2_CT*_x_*, V_4_C_3_T*_x_*, Nb_2_CT*_x_*, and Nb_4_C_3_T*_x_*) and Scalable Production of Ti_3_C_2_T*_x_*

To validate the versatility of the molecular intercalant strategy for other MXenes, we applied this method to a series of NH_4_F–etching MAX phase systems. The XRD patterns and SEM images of the high–purity MAX phases (Mo_2_TiAlC_2_, V_2_AlC, V_4_AlC_3_, Nb_2_AlC and Nb_4_AlC_3_) annealed by high–temperature solid–state reaction are shown in Figures S42–S46. These precursors were then converted to the corresponding MXenes (Mo_2_TiC_2_T*_x_*, V_2_CT*_x_*, V_4_C_3_T*_x_*, Nb_2_CT*_x_*, and Nb_4_C_3_T*_x_*) using the NH_4_F+thiourea system. XRD patterns (Figure 4a) and SEM images (Figure 4b–f) show shifts of the 002 diffraction peaks toward lower angles and the appearance of accordion‐like morphologies, respectively, indicating the successful formation of MXene. EDS elemental maps (Figures S47–S51) and XPS results (Figure 4g–k) reveal an absence of Al in the resulting MXenes, further confirming the completeness of the etching process. High–resolution XPS spectra (Figures S52–S56 and Tables S6–S10) show that different MXenes possess distinct atomic ratios of their surface functional groups (e.g., C–M–O(H), C–M–F, and C–M–S). Previous studies have demonstrated that conventional etching routes exhibit poor efficiency and lower yield for many MXenes (Table S11), providing further evidence (Figure 4l) for the effectiveness of the low–temperature molten salt route for Al–based MXenes. For the aforementioned small molecules (oxalic acid, acetamide, propionamide, and glycine) that cause over–etching in Ti_3_AlC_2_ MAX, the more corrosion–resistant Mo_2_TiAlC_2_ can still be successfully converted into Mo_2_TiC_2_T*_x_* MXene (Figures S57–S61), owing to the presence of exposed outer Mo atoms. These results indicate that optimizing the etching temperature and duration for Ti_3_AlC_2_ may provide a viable strategy for the controlled synthesis of Ti_3_C_2_T*_x_* MXene, despite the fact that treatments with oxalic acid, acetamide, propionamide, and glycine lead to over–etching, as discussed above. The molecular diversity and green chemistry nature of small–molecule intercalation provide an unexplored parameter space for optimizing MXene etching strategy and precisely tuning their interlayer spacing.

**FIGURE 4:**
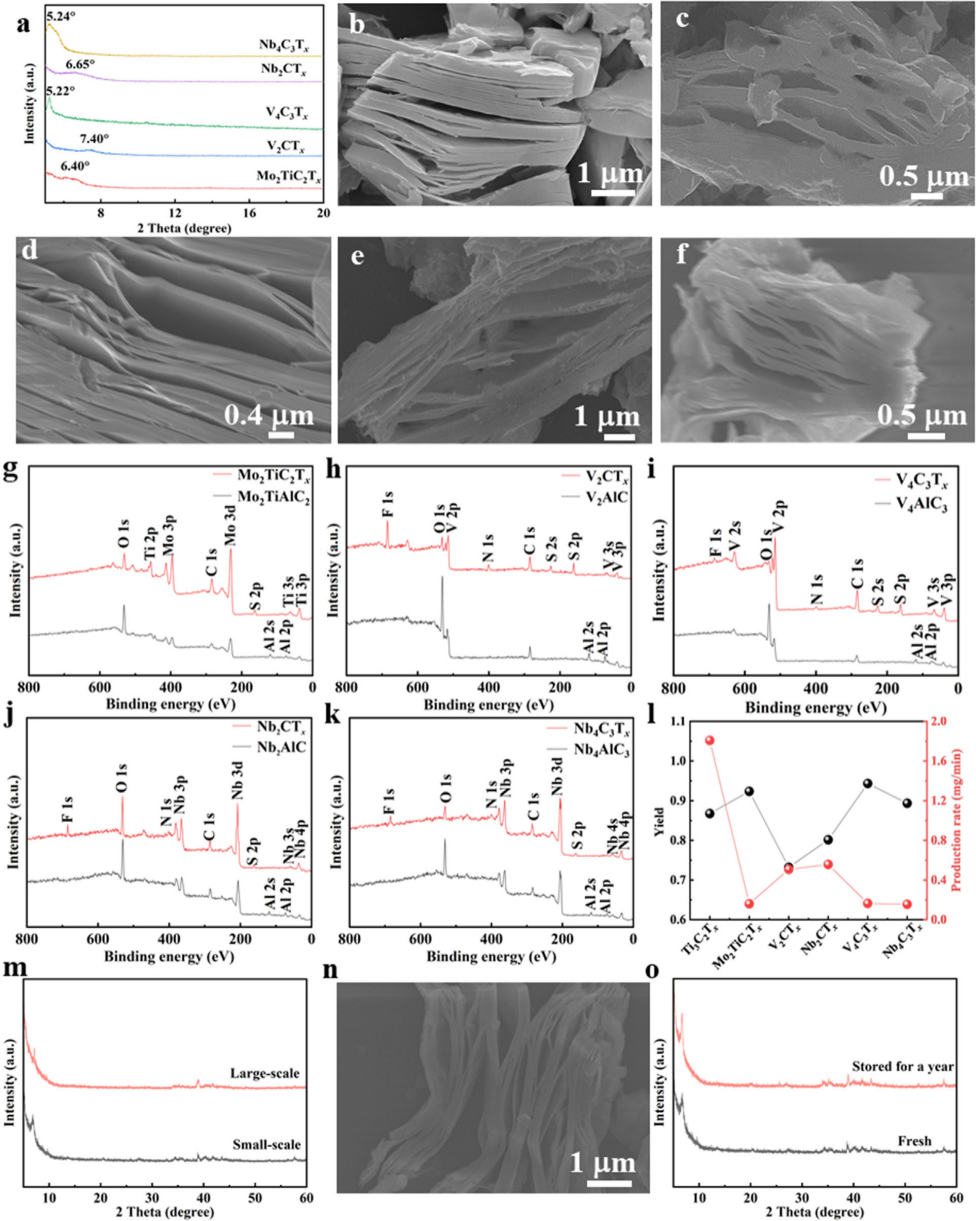
Experimental verification and characterization of small–molecule intercalation into five other MXenes (Mo_2_TiC_2_T*_x_*, V_2_CT*_x_*, V_4_C_3_T*_x_*, Nb_2_CT*_x_*, and Nb_4_C_3_T*_x_*) under low–temperature molten–salt conditions. (a) XRD patterns of the five MXenes etched using NH_4_F + thiourea. SEM images of (b) Mo_2_TiC_2_T*_x_*, (c) V_2_CT*_x_*, (d) V_4_C_3_T*_x_*, (e) Nb_2_CT*_x_*, and (f) Nb_4_C_3_T*_x_*. XPS survey spectra of (g) Mo_2_TiAlC_2_ and Mo_2_TiC_2_T*_x_*, (h) V_2_AlC and V_2_CT*_x_*, (i) V_4_AlC_3_ and V_4_C_3_T*_x_*, (j) Nb_2_AlC and Nb_2_CT*_x_*, and (k) Nb_4_AlC_3_ and Nb_4_C_3_T*_x_*. (l) The yields of small–batch MXene synthesis. (m) XRD patterns and (n) SEM image of the Ti_3_C_2_T*_x_* MXene prepared by large–scale synthesis. (o) XRD patterns of Ti_3_C_2_T*_x_* MXene after long–term storage for one year.

To further assess the reliability and practicality of large–scale production, we carried out a scaled–up synthesis of Ti_3_C_2_T*_x_* based on 6 g of the MAX phase precursor. As shown in Figure S62, more than 5.37 g of Ti_3_C_2_T*_x_* is successfully synthesized. The XRD patterns in Figure 4m also show that the (002) peak of the scaled–up Ti_3_C_2_T*_x_* MXene matches that of the small–scale product. The SEM image (Figure 4n) and EDS mappings (Figure S63) indicate that the large–scale Ti_3_C_2_T*_x_* retains the typical accordion–like morphology and exhibits effective Al removal. Compared with the small–scale experiment, the production rate (44.75 mg min^−1^) increases by nearly 25–fold, while the yield (∼89.5%) remains almost unchanged. Moreover, few–layer MXene was successfully produced by simple sonication (Figure S64), eliminating the need for any additional intercalants. The strategy affords Ti_3_C_2_T*_x_* MXene in a high and competitive isolated yield, while providing an alternative processing route to the established MILD method [51]. Notably, MXenes synthesized via this lowtemperature molten–salt method also exhibit exceptional environmental stability. When Ti_3_C_2_T*_x_* MXene is stored in a glass vial for one year without inert gas protection, the XRD patterns (Figure 4o) show no discernible changes, demonstrating outstanding long–term structural integrity and oxidation resistance. Moreover, the lower synthesis temperature and the simplified experimental conditions (Table S11) endow this method with strong potential for commercial scalability. Tables S12 and S13 compare the costs of etching agents and intercalation molecules across different etching methods, highlighting our method as a potentially cost–effective option based on raw–reagent prices.

Nevertheless, several aspects of the present study require further clarification. First, the molecular dimensions extracted from solid–state crystal structures serve only as approximate empirical descriptors and cannot fully capture the dynamic conformations, molecular orientations, or effective occupied volumes of guest species under molten–salt and confined MXene interlayer conditions. Second, the specific contributions of molecule–derived species generated through partial decomposition or protonation have not yet been directly identified. Future work will therefore combine in situ characterization, molecular dynamics simulations, and first–principles calculations to further clarify the effective molecular dimensions and molecule–surface interactions under realistic molten–salt etching conditions.

### 2.6 Electrocatalytic HER Performance

We subsequently compared the electrocatalytic hydrogen evolution performance of three representative MXenes (Ti_3_C_2_T*_x_*, Mo_2_TiC_2_T*_x_*, and V_4_C_3_T*_x_*) synthesized via low–temperature molten salt methods. Among them, Mo_2_TiC_2_T*_x_* MXene exhibits a markedly lower overpotential at 10 mA cm^−2^ (*η*_10_ = 251.6 mV) in 0.5 M H_2_SO_4_ (Figure 5a and Table S14) than Ti_3_C_2_T*_x_* (> 320.0 mV) and V_4_C_3_T*_x_* (304.6 mV), showing activity closest to that of the commercial Pt/C catalyst. The Tafel slopes derived from the polarization curves (Figure 5b and Table S14) show that Mo_2_TiC_2_T*_x_* MXene possesses a lower slope of 80.6 mV dec^−1^. The double–layer capacitance (C_dl_) (Figures 5c and S65) indirectly reflects the catalyst's electrochemically active surface area (ECSA). Although Mo_2_TiC_2_T_x_ does not show the highest C_dl_, it exhibits the best HER activity. The charge–transfer resistance (R_ct_) during HER was assessed by electrochemical impedance spectroscopy (EIS) (Figure 5d). According to the fitted equivalent circuit, Mo_2_TiC_2_T*_x_* MXene exhibits a smaller R_ct_ value (Table S15) compared to the Ti_3_C_2_T*_x_* (53.9 Ω) and V_4_C_3_T*_x_* (30.9 Ω). These results indicate that the superior HER performance of Mo_2_TiC_2_T_x_ is not governed solely by the ECSA, but is mainly associated with its lower charge–transfer resistance and enhanced intrinsic activity arising from favorable surface chemistry. We also conducted cycling–stability tests to evaluate the long–term durability of the catalysts. As shown in Figure 5e, Mo_2_TiC_2_T*_x_* MXene retains its catalytic activity after 24 h, exhibiting negligible changes in polarization curves (Figure 5f). To further assess pH–dependent activity, the catalytic performance was also evaluated in 1.0 M KOH (Figure S66). Notably, the Mo_2_TiC_2_T*_x_* MXene catalyst also demonstrates excellent HER activity in alkaline media, delivering a low overpotential of 321 mV at 10 mA cm^−2^ and a small Tafel slope of 121 mV dec^−1^, outperforming Ti_3_C_2_T*_x_* (> 575 mV, 356 mV dec^−1^) and V_4_C_3_T*_x_* (485 mV, 256 mV dec^−1^). This superior catalytic activity may be attributed to the presence of catalytically active Mo–based basal planes, highlighting Mo_2_TiC_2_T*_x_* as a promising HER electrocatalyst. The results show that the HER performance of the MXenes synthesized by our method is comparable to that of MXenes prepared using concentrated HF etching (Table S16), demonstrating the effectiveness of the present molten–salt etching strategy. For the molten–salt–etched MXenes, the F–termination coverage decreases in the order Ti_3_C_2_T*_x_* > V_4_C_3_T*_x_* > Mo_2_TiC_2_T*_x_*, with values of 42.46%, 9.67%, and 2.64%, respectively (Tables S5–S7). Correspondingly, their HER activity shows an increasing tendency with decreasing F–termination coverage, suggesting that surface F content may be one of the factors influencing HER performance.

**FIGURE 5:**
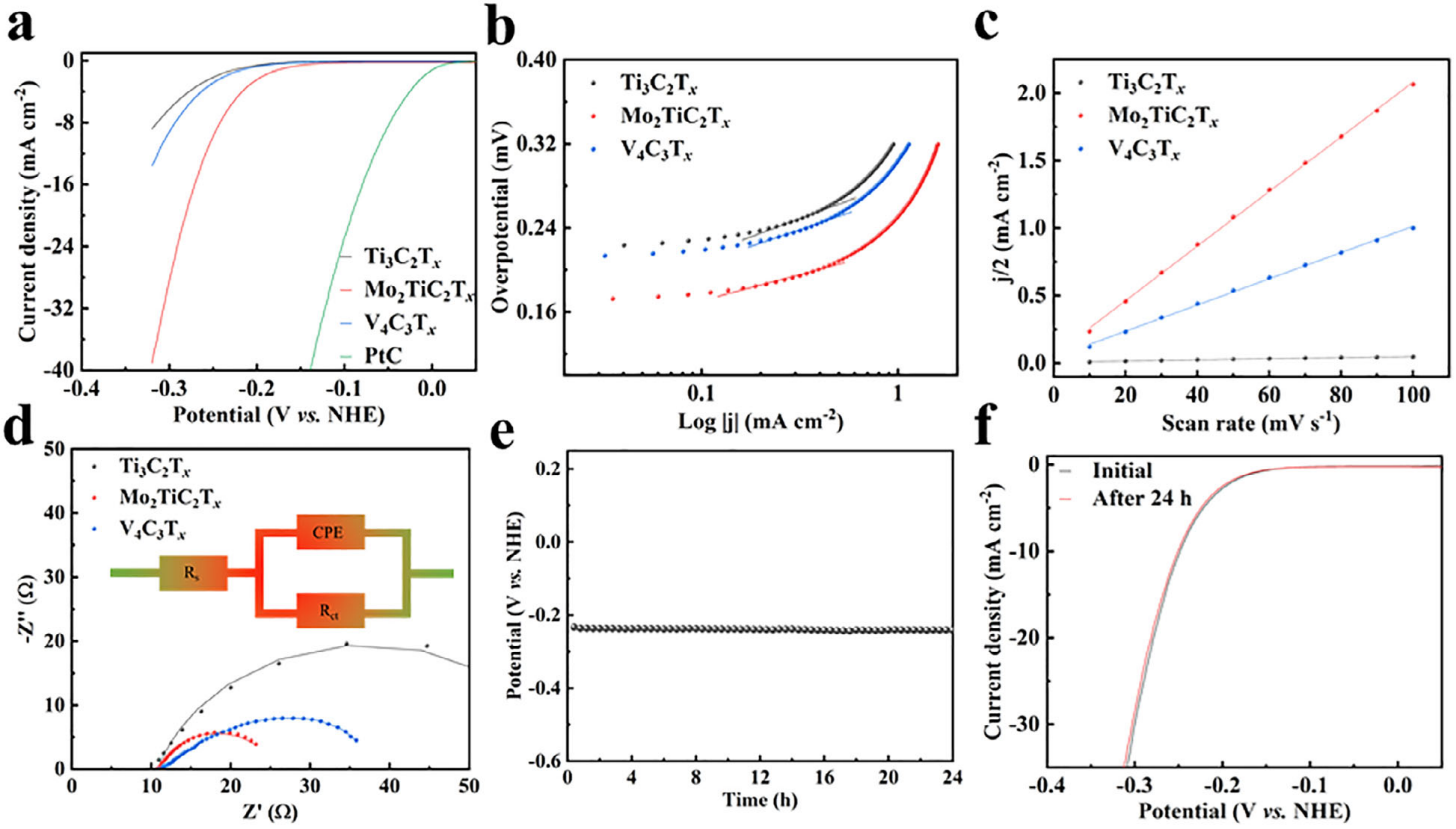
Electrocatalytic HER activity of three MXenes (Ti_3_C_2_T*_x_*, Mo_2_TiC_2_T*_x_*, and V_4_C_3_T*_x_*). (a) LSV curves of three MXenes and commercial Pt/C. (b) Tafel curves, (c) C_dl_ curves, and (d) EIS of the three MXenes. (e) Long–term stability of Mo_2_TiC_2_T*_x_* for HER at the current density of 10 mA cm^−2^. (f) LSV curves of Mo_2_TiC_2_T*_x_* before and after 24 h HER.

In conclusion, we present a high–yield MXene synthesis method that is safer, operationally simpler, and enables markedly faster access to exfoliated layered structures. This work provides a universal molecular–size criterion for assessing the compatibility of guest intercalants within MXene hosts. As the library of compatible intercalants expands, future research can harness structurally diverse small molecules to precisely tailor the electronic structure of MXene. This strategy opens new avenues for enhancing MXene performance across a broad range of applications, including energy storage, sensors, and environmental remediation.

## 3 Methods

### 3.1 Synthesis of MAX

Six MAX phases (Ti_3_AlC_2_, Mo_2_TiAlC_2_, V_2_AlC, V_4_AlC_3_, Nb_2_AlC, and Nb_4_AlC_3_) were synthesized via high–temperature solid–state routes. Stoichiometric amounts of high–purity precursor powders were thoroughly homogenized and sintered under an inert atmosphere (1350–1600°C) to complete the reaction. Experimental details are provided in the Supporting Information.

### 3.2 Ammonium Halide Etching (NH_4_X) of MAX Phases

250 mg Ti_3_AlC_2_ and 1.6 g NH_4_F were slowly added to a 100 mL PTFE–lined autoclave and gently shaken to homogenize. Then, the autoclave was placed in an oven at 200°C for 2 h to facilitate the etching reaction. After natural cooling to room temperature, the solid was repeatedly washed with hydrochloric acid, deionized water, and anhydrous ethanol until the pH was near neutral. The resulting powders were then vacuum–filtered and vacuum–dried at 60°C for 24 h. Other ammonium halides (e.g., NH_4_Cl, NH_4_Br, NH_4_I) can be used in the same manner as NH_4_F, without further adjustments to the procedure. Caution: NH_4_F is a hazardous fluoride‐containing reagent and may generate NH_3_, HF, and/or other volatile fluorine–containing species upon heating. Therefore, all operations involving NH_4_F and post–reaction products should be conducted in a well–ventilated fume hood using appropriate personal protective equipment.

### 3.3 Solvent‐Free Synthesis of Multilayer MXenes

Multilayer MXene can be rapidly produced by reacting MAX precursor with NH_4_F in the presence of small–molecule molten–salt media. As a representative protocol using thiourea as the small molecule for Ti_3_C_2_T*_x_* MXene synthesis, 250 mg of Ti_3_AlC_2_ MAX powders, 1.5 g NH_4_F, and 1.5 g thiourea were thoroughly homogenized in a PTFE–lined autoclave and held in an oven at 200°C for 2 h. After natural cooling to room temperature, the solid was washed repeatedly with hydrochloric acid, deionized water, and anhydrous ethanol until the pH value of the filtrate was near neutral. The product was then vacuum–filtered and vacuum–dried at 60°C for 24 h to afford the multilayer Ti_3_C_2_T*_x_* MXene.

### 3.4 Synthesis of Few‐Layer Ti_3_C_2_T*_x_* MXene

The obtained multilayer Ti_3_C_2_T*_x_* MXene was dispersed in 100 mL of ultrapure water and ultrasonicated for 1 h to obtain few–layer Ti_3_C_2_T*_x_* MXene nanosheets. A 20 mL aliquot of the dispersion was filtered through an mixed cellulose esters (MCE) membrane under vacuum and dried at room temperature to fabricate a free–standing MXene film.

### 3.5 Optimized Synthesis of Large‐Scale Ti_3_C_2_T*_x_* MXene

6 g of Ti_3_AlC_2_ MAX powders, 6 g NH_4_F, and 6 g thiourea were thoroughly homogenized in a PTFE–lined autoclave and held in an oven at 200°C for 2 h. After natural cooling to room temperature, the solid was washed repeatedly with hydrochloric acid, deionized water, and anhydrous ethanol until the pH value of the filtrate was near neutral. The product was then vacuum–filtered and vacuum–dried at 60°C for 24 h to obtain the Ti_3_C_2_T*_x_* MXene.

### 3.6 Materials Characterizations

Micromorphological properties of the samples were observed using scanning electron microscopy (SEM, JSM6390, JEOL, Japan) and transmission electron microscopy (TEM, JEM–2010, JEOL, Japan). Microstructural properties of the samples were recorded using an X–ray diffractometer (XRD, D/max 2200, Rigaku, Japan) using Cu Kα radiation (λ = 0.15406 nm, 40 kV, 10 mA). Elemental analysis of the samples was performed using an X–ray photoelectron spectroscopy (XPS, ESCALAB 250, VG Thermo, USA) using Al Kα radiation. Raman spectra of the samples were recorded using a LabRam HR Evolution Raman spectrometer with a Nd:YAG laser (*λ* = 532 nm). Fourier transform infrared spectra were collected with an instrument in ATR mode (Nicolet iS50, Bruker).

### 3.7 Electrochemical HER Measurements

The electrochemical HER performance of the prepared catalysts was measured by a three–electrode configuration in 0.5 M H_2_SO_4_ and 1.0 M KOH on a CHI660E electrochemical workstation. According to Nernst equation: E versus RHE (V) = E versus SCE (V) + 0.05916 × pH + 0.244 (V) (pH = 0.6), E versus RHE (V) = E versus Hg/HgO (V) + 0.05916 × pH + 0.098 (V) (pH = 14), the recorded potential should be corrected by the reversible hydrogen electrode. The working electrode was a glassy carbon electrode (GCE, 3.0 mm in diameter) loaded with 0.2 mg/cm^2^ of catalytically active materials. Carbon rod electrode and a saturated calomel electrode (SCE) were selected as the counter electrode and reference electrode, respectively. Typically, the catalyst slurry was prepared as follows: 4 mg of active material, 800 µL of ethanol, 185 µL of deionized water, and 15 µL of Nafion were ultrasonicated for 40 min to form a homogeneous dispersion. Then 3.6 µL of catalyst slurry was drop–cast onto the GCE surface and dried at room temperature.

### 3.8 Computational Details

All the calculations were carried out using the Vienna Ab initio Simulation Package (VASP) with the frozen–core all–electron projector–augmented wave (PAW) method [54, 55]. The generalized gradient approximation (GGA) proposed by Perdew–Burke–Ernzerhof (PBE) was selected for the exchange–correlation potential [56]. Grimme's method (DFT–D3) was included during the surface adsorption to better evaluate the van der Waals interaction [57]. The cut–off energy for the plane wave was set to 550 eV. The k–space integration was done with Γ–point mesh in the 2D Brillouin zones. The thresholds were 10^−5^ eV and 0.03 eV/Å for electronic and ionic relaxations, respectively.

### 3.9 Additional Methods

For details describing chemical reagents and material preparation (including the synthesis of MXenes such as Mo_2_TiC_2_T*_x_*, V_2_CT*_x_*, V_4_C_3_T*_x_*, Nb_2_CT*_x_*, and Nb_4_C_3_T*_x_*), please see Supplementary Information.

## Author Contributions

W.Q., Y.A., and Y.Y. supervised the project. M.S. and X.B. conceived the idea and designed the experiments. M.S., X.B., and W.Q. discussed and proposed the mechanism. M.S. performed the theoretical calculations and the experiments. X.L. and Y.Z. performed some characterization. All authors discussed the results and commented on the paper.

## Conflicts of Interest

The authors declare no conflicts of interest.

## Supporting information




**Supporting File**: adma74797‐sup‐0001‐SuppMat.docx.

## Data Availability

The data that support the findings of this study are available from the corresponding author upon reasonable request.
