# Supplementary material for: Spatial‐Compatibility‐Assisted Molecular Intercalation in MXenes
Source: Adv Mater. 2026 Sep 4;38(60):e74797. doi: 10.1002/adma.74797 (PMC13712195; doi:10.1002/adma.74797)
Supplement: Supplementary file 1 [file ADMA-38-e74797-s001.docx]

**Spatial-compatibility-assisted molecular intercalation in MXenes**

**Minhao Sheng ^a, b^, Xiaoqing Bin ^a^, Xiangyang Liu ^b^, Yingji Zhao ^b^, Wenxiu Que ^a,^ *, Yusuke Asakura ^b, *^, Yusuke Yamauchi ^b, c, d, *^**

^a^ Electronic Materials Research Laboratory, Key Laboratory of the Ministry of Education, International Center for Dielectric Research, Shaanxi Engineering Research Center of Advanced Energy Materials and Devices, School of Electronic Science and Engineering, Xi’an Jiaotong University, Xi’an 710049, P. R. China.

^b^ Department of Materials Process Engineering, Graduate School of Engineering, Nagoya University, Nagoya 464-8603, Japan;

^c^ Australian Institute for Bioengineering and Nanotechnology (AIBN), The University of Queensland, Brisbane, Queensland 4072, Australia

^d^ Department of Chemical and Biomolecular Engineering, Yonsei University, Seoul 03722, South Korea.

* [wxque@mail.xjtu.edu.cn](mailto:wxque@mail.xjtu.edu.cn);

[asa.y@nagoya-u.jp](mailto:asa.y@nagoya-u.jp); y.yamauchi@uq.edu.au

**This PDF file includes:**

Materials and Methods

Supplementary Text

Figs. S1 to S66

Tables S1 to S16

Full Reference List

**Materials and Methods**

**1.1	Materials**

Ammonium fluoride (NH_4_F, 98%), ammonium chloride (NH_4_Cl, 99%), ammonium bromide (NH_4_Br, 98%), and ammonium iodide (NH_4_I, 99%) were of analytical grade and obtained from FUJIFILM Wako Pure Chemical Corporation. Molybdenum powder (Mo, 2 µm mesh particle size, 99.99%), titanium powder (Ti, 300 mesh, 99.99%), niobium powder (Nb, 400 mesh, 99.5%), vanadium powder (V, 400 mesh, 99.8%), titanium carbide (TiC, 2-4 µm particle size, 99.5%), aluminum (Al, 400 mesh, 99.9%), and graphite powder (C, 99.95%) were purchased from Aladdin. Urea (CO(NH_2_)_2_, 99.8%), thiourea (CS(NH_2_)_2_, 99%), oxalic acid (C_2_H_2_O_4_, 99.5%), citric acid (C_6_H_8_O_7_, 99.8%), uric acid (C_5_H_6_N_4_O_3_, 99.9%), cetyltrimethylammonium bromide (C_19_H_42_NBr, 99.9%), and cyanuric acid (C_3_H_3_N_3_O_3_, 99.5%) were purchased from Sigma-Aldrich. Acetamide (C_2_H_5_NO, 99.5%), propionamide (C_3_H_7_NO, 99.5%), glycine (C_2_H_5_NO_2_, 99.9%), pyrazine (C_4_H_4_N_2_, 99.5%), and ethyl alcohol (C_2_H_5_OH, 99.5%) were purchased from FUJIFILM Wako Pure Chemical Corporation. All chemical reagents were directly used as purchased without further purification.

**1.2	Preparation of precursor MAX phases.**

High-purity Ti_3_AlC_2_, Mo_2_TiAlC_2_, V_2_AlC, V_4_AlC_3_, Nb_2_AlC, and Nb_4_AlC_3_ MAX-phase powders were annealed through high-temperature solid-state reaction under an argon atmosphere. The detailed experimental procedures are as follows.

Ti_3_AlC_2_ was prepared using commercial powders (TiC, Al, and Ti). The molar ratio of Ti/TiC/Al was 1:2:1.2. The powders were mixed with ethyl alcohol in an agate mortar and then dried in an oven at 60°C for 24 h. Subsequently, the powder mixtures were prepared by high-temperature solid-state reactions in an atmosphere furnace at 1350°C and held for 2 h under flowing argon gas. After being cooled, the sintered bulk was ground and the ground product was then sieved through a 400-mesh sieve to obtain particles smaller than 38 µm. For other MAX phases, the experimental conditions for these reactions were consistent with those employed in the preceding procedures.

For Mo_2_TiAlC_2_, the molar ratio of Mo/Ti/Al/C was 2:1:1.2:1, and the powder mixtures were annealed in an atmosphere furnace at 1600°C and held for 4 h. For V­­_2_AlC, the molar ratio of V/Al/C was 2:1.2:0.9, and the powder mixtures were annealed in an atmosphere furnace at 1500°C and held for 4 h. For V­­_4_AlC_3_, the molar ratio of V/Al/C was 4:1.5:2.7, and the powder mixtures were annealed in an atmosphere furnace at 1600°C and held for 4 h. For Nb_2_AlC, the molar ratio of Nb/Al/C was 2:1.2:0.9, and the powder mixtures were annealed in an atmosphere furnace at 1500°C and held for 4 h. For Nb­­_4_AlC_3_, the molar ratio of Nb/Al/C was 4:1.5:2.7, and the powder mixtures were annealed in an atmosphere furnace at 1600°C and held for 4 h.

**1.3	Preparation of other** **MXenes (Mo_2_TiC_2_T*_x_*, V_2_CT*_x_*, V_4_C_3_T*_x_*, Nb_2_CT*_x_*, and Nb_4_C_3_T*_x_*)**

For Mo_2_TiC_2_T*_x_* MXene, 250 mg of Mo_2_TiAlC_2_ MAX powders, 1.5 g NH_4_F, and 1.5 g thiourea were thoroughly homogenized in a PTFE-lined autoclave and held in an oven at 200°C for 24 h. After natural cooling to room temperature, the solid was washed repeatedly with hydrochloric acid, deionized water, and anhydrous ethanol until the filtrate was near neutral. The product was then vacuum-filtered and vacuum-dried at 60°C for 24 h to afford the multilayer Mo_2_TiC_2_T*_x_* MXene.

For V_2_CT*_x_* MXene, the precursor was replaced with V_2_AlC, and the etching time was kept at 6 hours. For V_4_C_3_T*_x_* MXene, the precursor was replaced with V_4_AlC_3_, and the etching time was kept at 24 hours. For Nb_2_CT*_x_* MXene, the precursor was replaced with Nb_2_AlC, and the etching time was kept at 6 hours. For Nb_4_C_3_T*_x_* MXene, the precursor was replaced with Nb_4_AlC_3_, and the etching time was kept at 24 hours.

**1.4	Guidelines for preparing MXenes from NH_4_X (X=F, Cl, Br, I) molten salt etching route**

The Gibbs free energy (ΔG_r_) was calculated to guide the etching of various MAX phase precursors in NH_4_X for the preparation of MXene materials. Generally, the covalent M-C bonding in the MAX phase is very strong, while the M-A bonding is much weaker, facilitating selective etching of the A-layers. Hence, we assume that the M-C bonding remains unchanged during the etching reaction. We used elemental Al as a simplified model species representing the Al layers in the MAX phase.

The etching reaction was simplified as equation (1):

Al + 3 NH_4_X = AlX_3_(g) + 1.5 H_2_(g) + 3 NH_3_(g)	(1)

The ΔG_r_ between Al element and NH_4_X was calculated by HSC Chemistry software (HSC 6.0). Specifically, the calculation equation of ΔG_r_ is as follows:

ΔG_r_ = ΔH_r_ − TΔS_r_	(2)

For the equation (2), ΔH_r_ stands for enthalpy change, ∆S_r_ is entropy change, and T is the reaction temperature. ΔH_r_ and ΔS_r_ can be obtained from the HSC software.

**1.5	M-Al bond length and interlayer spacing**

Based on the crystallographic data of the MAX phase (hexagonal lattice), the M-Al bond length can be directly calculated from the lattice parameters and the fractional coordinates of neighboring M and Al atoms, denoted as D_M−Al_. The interlayer spacing of the derived MXene is estimated from the MAX structure as the distance along the c axis between the two nearest-neighbor transition-metal atoms (M atoms) across the removed Al layer, defined as D_inter_.

**1.6	Three-dimensional space occupied by individual molecular crystals (X, Y, Z)**

The atomic coordinates and unit cell parameters provided in the CIF file for each molecular crystal allow the conversion of fractional coordinates into Cartesian coordinates, determining the actual positions of the atoms in three-dimensional space. Next, an individual molecule is extracted from the unit cell. For example, in the case of thiourea, the CS(NH_2_)_2_ molecule can be isolated. By analyzing the distances between atoms and the molecular shape, the three-dimensional space (X, Y, Z) occupied by the molecule within the crystal can be calculated.

**Supplementary Text**

Fig. S1. Ti–Al bond length calculated based on the crystal structure^1^ of Ti_3_AlC_2_.

Fig. S2. Mo–Al bond length calculated based on the crystal structure of Mo_2_TiAlC_2_. The crystal structure information was obtained from [*https://materials.springer.com/isp/crystallographic/docs/sd*](https://materials.springer.com/isp/crystallographic/docs/sd) *_1643284.*

Fig. S3. Nb–Al bond length calculated based on the crystal structure^2^ of Nb_2_AlC.

Fig. S4. Nb–Al bond length calculated based on the crystal structure^3^ of Nb_4_AlC_3_.

Fig. S5. V–Al bond length calculated based on the crystal structure^4^ of V_2_AlC.

Fig. S6. V–Al bond length calculated based on the crystal structure^5^ of V_4_AlC_3_.

Fig. S7. SEM images of Ti_3_AlC_2_.

Fig. S8. SEM images of Ti_3_AlC_2_ after NH_4_F–etching. Following NH_4_F etching, two distinct morphologies are observed: (i) an undulating, accordion-like lamellar architecture diagnostic of MXene and (ii) dense, blocky particles consistent with the parent MAX phase. The coexistence of these features indicates that, under the present etching protocol, conversion is incomplete and a residual MAX fraction remains.

Fig. S9. (a) The elemental maps and (b) EDS spectrum of Ti_3_AlC_2_. The Si peak originates from the Si substrate rather than the sample itself.

Fig. S10. The elemental maps of Ti_3_AlC_2_ after NH_4_F–etching. The uneven distribution of Ti and Al suggests that Al preferentially dissolves under the etching of ammonium fluoride.

Fig. S11. SEM images of Ti_3_AlC_2_ after NH_4_Cl–etching. The rough surface suggests that Cl^-^ ions have difficulty penetrating the Al sites in Ti_3_AlC_2_ and are limited to the MAX surface.

Fig. S12. SEM images of Ti_3_AlC_2_ after NH_4_Br–etching. The rough surface suggests that Br^-^ ions have difficulty penetrating the Al sites in Ti_3_AlC_2_ and are limited to the MAX surface.

Fig. S13. SEM images of Ti_3_AlC_2_ after NH_4_I–etching. The rough surface suggests that I^-^ ions have difficulty penetrating the Al sites in Ti_3_AlC_2_ and are limited to the MAX surface.

Fig. S14. The elemental maps of Ti_3_AlC_2_ after NH_4_Cl–etching. The uniform distribution of Ti and Al indicates the presence of the MAX phase's main structure.

Fig. S15. The elemental maps of Ti_3_AlC_2_ after NH_4_Br–etching. The uniform distribution of Ti and Al indicates the presence of the MAX phase's main structure.

Fig. S16. The elemental maps of Ti_3_AlC_2_ after NH_4_I–etching. The uniform distribution of Ti and Al indicates the presence of the MAX phase's main structure.

Fig. S17. EDS spectra of Ti_3_AlC_2_ after NH_4_X (X=F, Cl, Br, I)–etching. The significantly increased Ti/Al atomic ratio in the NH_4_F etching products confirms the removal of Al, while the decreased Ti/Al atomic ratio in the NH_4_Cl, NH_4_Br, and NH_4_I etching products compared to the original Ti_3_AlC_2_ MAX suggests that the Ti on the MAX surface is more easily attacked by Cl^-^, Br^-^, and I^-^ ions.

Fig. S18. XRD patterns of Ti_3_AlC_2_ and ammonium halide etching products. The XRD pattern after NH_4_F etching shows a significant reduction in intensity compared to the original MAX phase and other halide etching products, indirectly confirming the dissolution of Al.

Fig. S19. Crystal structure of urea. The corresponding Wyckoff positions and fractional coordinates of C, O, N, and H atoms are summarized in the table. The crystal structure is obtained from the Crystallography Open Database (COD 1008776).

Fig. S20. Crystal structure of thiourea. The corresponding Wyckoff positions and fractional coordinates of C, S, N, and H atoms are summarized in the table. The crystal structure is obtained from the Crystallography Open Database (COD 8103728).

**Fig. S21.** Crystal structure of oxalic acid. The corresponding Wyckoff positions and fractional coordinates of C, O, and H atoms are summarized in the table. The crystal structure is obtained from the Crystallography Open Database (COD 4116148).

**Fig. S22.** Crystal structure of cyanuric acid. The corresponding Wyckoff positions and fractional coordinates of C, O, N, and H atoms are summarized in the table. The crystal structure is obtained from the Crystallography Open Database (COD 2106069).

**Fig. S23.** Crystal structure of uric acid. The corresponding Wyckoff positions and fractional coordinates of C, O, N, and H atoms are summarized in the table. The crystal structure is obtained from the Crystallography Open Database (COD 9011061).

**Fig. S24.** Crystal structure of citric acid. The corresponding Wyckoff positions and fractional coordinates of C, O, and H atoms are summarized in the table. The crystal structure is obtained from the Crystallography Open Database (COD 5000063).

**Fig. S25.** Crystal structure of cetyltrimethylammonium bromide. The corresponding Wyckoff positions and fractional coordinates of C, Br, N, and H atoms are summarized in the table. The crystal structure is obtained from the Crystallography Open Database (COD 7206057).

Fig. S26-1. SEM images of Ti_3_AlC_2_ after NH_4_F+urea at 200 °C for 2 h.

**Fig. S26-2**. The elemental maps of Ti_3_AlC_2_ after NH_4_F+urea at 200 °C for 2 h.

**Fig. S26-3.** EDS spectrum of Ti_3_AlC_2_ after NH_4_F+urea at 200 °C for 2 h.

Fig. S27-1. SEM images of Ti_3_AlC_2_ after NH_4_F+thiourea at 200 °C for 2 h.

**Fig. S27-2.** The elemental maps of Ti_3_AlC_2_ after NH_4_F+thiourea at 200 °C for 2 h.

**Fig. S27-3.** EDS spectrum of Ti_3_AlC_2_ after NH_4_F+thiourea at 200 °C for 2 h.

Fig. S28. High-resolution (a) Ti 2p, (b) C 1s, (c) N 1s, (d) O 1s, (e) F 1s, and (f) S 2p XPS spectra of Ti_3_C_2_T*_x_*. The N 1s spectrum was fitted with three components assigned to quaternary N (N-Q), pyridinic N (N-1), and Ti–N nitride species^6,7^.

Fig. S29. XRD pattern of Ti_3_AlC_2_ after NH_4_F+oxalic acid etching at 200 °C for 2 h.

Fig. S30. SEM images of Ti_3_AlC_2_ treated with NH_4_F+oxalic acid at 200 °C for 2 h.

Fig. S31. The elemental maps of Ti_3_AlC_2_ treated with NH_4_F+oxalic acid at 200 °C for 2 h.

Fig. S32. Crystal structure of acetamide. The corresponding Wyckoff positions and fractional coordinates of C, H, N, and O atoms are summarized in the table. The red dashed area represents that the three-dimensional spatial dimensions of individual small molecules. The crystal structure is obtained from the Crystallography Open Database (COD 9007523).

Fig. S33. Crystal structure of propionamide. The corresponding Wyckoff positions and fractional coordinates of C, H, N, and O atoms are summarized in the table. The red dashed area represents that the three-dimensional spatial dimensions of individual small molecules. The crystal structure is obtained from the Cambridge Structural Database (CSD ZZZKAY04).

Fig. S34. Crystal structure of glycine. The corresponding Wyckoff positions and fractional coordinates of C, H, N, and O atoms are summarized in the table. The red dashed area represents that the three-dimensional spatial dimensions of individual small molecules. The crystal structure is obtained from the Crystallography Open Database (COD 2103309).

Fig. S35. XRD patterns of Ti_3_AlC_2_ treated with NH_4_F+small molecules (acetamide, propionamide, and glycine) etching at 200 °C for 2 h.

Fig. S36-1. SEM images of Ti_3_AlC_2_ after NH_4_F+acetamide etching at 200 °C for 2 h.

**Fig. S36-2.** The elemental maps of Ti_3_AlC_2_ after NH_4_F+acetamide etching at 200 °C for 2 h.

Fig. S37-1. SEM images of Ti_3_AlC_2_ treated with NH_4_F+propionamide at 200 °C for 2 h.

Fig. S37-2. The elemental maps of Ti_3_AlC_2_ treated with NH_4_F+propionamide at 200 °C for 2 h.

Fig. S38. SEM images of Ti_3_AlC_2_ treated with NH_4_F+**glycine** at 200 °C for 2 h.

Fig. S39. (a) XRD pattern of Ti_3_AlC_2_ after NH_4_F+urea etching at 200 °C for 6 h. (b) XRD pattern of Ti_3_AlC_2_ after NH_4_F+oxalic acid etching at 150 °C for 0.5 h. (c) XRD pattern of Ti_3_AlC_2_ after NH_4_F+acetamide and (d) NH_4_F+propionamide etching at 200 °C for 0.5 h.

Fig. S40-1. Crystal structure of butyramide. The corresponding Wyckoff positions and fractional coordinates of C, H, O, and N atoms are summarized in the table. The red dashed area represents that the three-dimensional spatial dimensions of individual small molecules. The crystal structure is obtained from the Crystallography Open Database (COD 277748).

Fig. S40-2. XRD pattern of Ti_3_AlC_2_ after NH_4_F+butyramide etching at 200 °C for 2 h.

Fig. S41-1. XRD patterns of Ti_3_AlC_2_ after NH_4_F+medium- and large-molecule etching at 200 °C for 2 h.

**Fig. S41-2.** SEM images of Ti_3_AlC_2_ treated with NH_4_F+(a1-a3) cyanuric acid, (b1-b3) uric acid, (c1-c3) citric acid, and (d1-d3) cetyltrimethylammonium bromide at 200 °C for 2 h.

Fig. S42-1. XRD pattern of Mo_2_TiAlC_2_.

Fig. S42-2. SEM image and the elemental maps of Mo_2_TiAlC_2_.

Fig. S43-1. XRD pattern of V_2_AlC.

Fig. S43-2. SEM image and the elemental maps of V_2_AlC.

Fig. S44-1. XRD pattern of V_4_AlC_3_.

Fig. S44-2. SEM image and the elemental maps of V_4_AlC_3_.

Fig. S45-1. XRD pattern of Nb_2_AlC.

Fig. S45-2. SEM image and the elemental maps of Nb_2_AlC.

Fig. S46-1. XRD pattern of Nb_4_AlC_3_.

Fig. S46-2. SEM image and the elemental maps of Nb_4_AlC_3_.

Fig. S47-1. SEM images and the elemental maps of Mo_2_TiAlC_2_ after NH_4_F+thiourea etching at 200 °C for 24 h.

**Fig. S47-2.** EDS spectrum of Mo_2_TiAlC_2_ after NH_4_F+thiourea etching at 200 °C for 24 h.

Fig. S48-1. SEM images and the elemental maps of V_2_AlC after NH_4_F+thiourea etching at 200 °C for 6 h.

**Fig. S48-2.** EDS spectrum of V_2_AlC after NH_4_F+thiourea etching at 200 °C for 6 h.

Fig. S49-1. SEM images and the elemental maps of V_4_AlC_3_ after NH_4_F+thiourea etching at 200 °C for 24 h.

Fig. S49-2. EDS spectrum of V_4_AlC_3_ after NH_4_F+thiourea etching at 200 °C for 24 h.

Fig. S50-1. SEM images and the elemental maps of Nb_2_AlC after NH_4_F+thiourea etching at 200 °C for 6 h.

Fig. S50-2. EDS spectrum of Nb_2_AlC after NH_4_F+thiourea etching at 200 °C for 6 h.

Fig. S51-1. SEM images and the elemental maps of Nb_4_AlC_3_ after NH_4_F+thiourea etching at 200 °C for 24 h.

Fig. S51-2. EDS spectrum of Nb_4_AlC_3_ after NH_4_F+thiourea etching at 200 °C for 24 h.

Fig. S52. High-resolution (a) Mo 3d, (b) Ti 2p, (c) C 1s, (d) N 1s, (e) O 1s, (f) F 1s, and (g) S 2p XPS spectra of Mo_2_TiC_2_T*_x_* after NH_4_F+thiourea etching at 200 °C for 24 h. The N 1s spectrum was fitted with two components assigned to pyrrolic N (N-2) and Mo–N nitride species^6,8^.

Fig. S53. High-resolution (a) V 2p, (b) C 1s, (c) N 1s, (d) O 1s, (e) F 1s, and (f) S 2p XPS spectra of V_2_CT*_x_* after NH_4_F+thiourea etching at 200 °C for 6 h.

Fig. S54. High-resolution (a) V 2p, (b) C 1s, (c) N 1s, (d) O 1s, (e) F 1s, and (f) S 2p XPS spectra of V_4_C_3_T*_x_* after NH_4_F+thiourea etching at 200 °C for 24 h.

Fig. S55. High-resolution (a) Nb 3d, (b) C 1s, (c) N 1s, (d) O 1s, (e) F 1s, and (f) S 2p XPS spectra of Nb_2_CT*_x_* after NH_4_F+thiourea etching at 200 °C for 6 h.

Fig. S56. High-resolution (a) Nb 3d, (b) C 1s, (c) N 1s, (d) O 1s, (e) F 1s, and (f) S 2p XPS spectra of Nb_4_C_3_T*_x_* after NH_4_F+thiourea etching at 200 °C for 24 h.

Fig. S57-1. SEM images and the elemental maps of Mo_2_TiAlC_2_ after NH_4_F+oxalic acid etching at 200 °C for 24 h.

Fig. S57-2. EDS spectrum of Mo_2_TiAlC_2_ after NH_4_F+oxalic acid etching at 200 °C for 24 h.

Fig. S58-1. SEM images and the elemental maps of Mo_2_TiAlC_2_ after NH_4_F+acetamide etching at 200 °C for 24 h.

Fig. S58-2. EDS spectrum of Mo_2_TiAlC_2_ after NH_4_F+acetamide etching at 200 °C for 24 h.

Fig. S59-1. SEM images and the elemental maps of Mo_2_TiAlC_2_ after NH_4_F+propionamide etching at 200 °C for 24 h.

**Fig. S59-2.** EDS spectrum of Mo_2_TiAlC_2_ after NH_4_F+propionamide etching at 200 °C for 24 h.

Fig. S60-1. SEM images and the elemental maps of Mo_2_TiAlC_2_ after NH_4_F+glycine etching at 200 °C for 24 h.

**Fig. S60-2.** EDS spectrum of Mo_2_TiAlC_2_ after NH_4_F+glycine etching at 200 °C for 24 h.

Fig. S61. XRD patterns of Mo_2_TiAlC_2_ after NH_4_F+small-molecule (oxalic acid, acetamide, propionamide, and glycine) etching at 200 °C for 24 h.

Fig. S62. Digital image of the Ti_3_C_2_T_x_ powder prepared by large-scale synthesis. The vessel shown in the figure is a 20 mL glass vial.

Fig. S63-1. SEM image and elemental mapping of the Ti_3_C_2_T*_x_* prepared by large-scale synthesis.

Fig. S63-2. EDS spectrum of Ti_3_C_2_T*_x_* prepared by large-scale synthesis.

Fig. S64. SEM images of few-layer Ti_3_C_2_T*_x_* MXene obtained from the scale-up MXene product via simple sonication.

Fig. S65. CV curves of (a) Ti_3_C_2_T*_x_*, (b) Mo_2_TiC_2_T*_x_*, and (c) V_4_C_3_T*_x_* at various scan rates in 0.5 M H_2_SO_4_.

Fig. S66. (a) LSV curves and (b) Tafel analysis of three MXenes in 1 M KOH.

Table S1. Gibbs free energy ΔG_r_ results of the reaction of NH_4_F with Al element in MAX phases.

| **T/°C** | **ΔH_r_/kcal mol^-1^** | **ΔS_r_/cal K^-1^ mol^-1^** | **ΔG_r_/kcal mol^-1^** |
| --- | --- | --- | --- |
| 0 | 10.46 | 192.89 | -42.23 |
| 40 | 10.41 | 192.73 | -49.94 |
| 80 | 10.22 | 192.14 | -57.64 |
| 120 | 9.87 | 192.22 | -65.70 |
| 160 | 9.37 | 190.02 | -72.93 |
| 200 | 8.72 | 188.59 | -80.51 |

Table S2. Gibbs free energy ΔG_r_ results of the reaction of NH_4_Cl with Al element in MAX phases.

| **T/°C** | **ΔH_r_/kcal mol^-1^** | **ΔS_r_/cal K^-1^ mol^-1^** | **ΔG_r_/kcal mol^-1^** |
| --- | --- | --- | --- |
| 0 | 52.24 | 186.56 | 1.28 |
| 40 | 52.67 | 184.61 | -5.14 |
| 80 | 51.96 | 182.49 | -12.48 |
| 120 | 51.11 | 180.22 | -19.74 |
| 160 | 50.12 | 177.82 | -26.90 |
| 200 | 46.45 | 169.77 | -33.87 |

Table S3. Gibbs free energy ΔG_r_ results of the reaction of NH_4_Br with Al element in MAX phases.

| **T/°C** | **ΔH_r_/kcal mol^-1^** | **ΔS_r_/cal K^-1^ mol^-1^** | **ΔG_r_/kcal mol^-1^** |
| --- | --- | --- | --- |
| 0 | 64.00 | 182.23 | 14.23 |
| 40 | 63.40 | 180.17 | 6.98 |
| 80 | 62.76 | 178.24 | -0.19 |
| 120 | 62.12 | 176.52 | -7.28 |
| 160 | 59.48 | 170.09 | -14.20 |
| 200 | 59.27 | 169.63 | -20.99 |

Table S4. Gibbs free energy ΔG_r_ results of the reaction of NH_4_I with Al element in MAX phases.

| **T/°C** | **ΔH_r_/kcal mol^-1^** | **ΔS_r_/cal K^-1^ mol^-1^** | **ΔG_r_/kcal mol^-1^** |
| --- | --- | --- | --- |
| 0 | 66.00 | 187.51 | 14.78 |
| 40 | 65.61 | 186.17 | 7.31 |
| 80 | 65.17 | 184.86 | -0.11 |
| 120 | 64.69 | 183.57 | -7.48 |
| 160 | 64.17 | 182.31 | -14.80 |
| 200 | 63.60 | 181.05 | -22.06 |

**Table S5-1.** Ti 2p high-resolution XPS fitting parameters of Ti_3_C_2_T*_x_* (NH_4_F+thiourea).

| **Peak** | **Position** | **Area** | **FWHM (eV)** |
| --- | --- | --- | --- |
| **C-Ti-T*_x_*** | 455.39 | 20897.70 | 1.02 |
|  | 461.02 | 8061.08 | 1.54 |
| **C-Ti^2+^** | 456.22 | 14239.10 | 1.00 |
|  | 461.73 | 6624.48 | 1.17 |
| **C-Ti^3+^** | 457.15 | 12115.61 | 1.27 |
|  | 462.73 | 5219.22 | 1.27 |
| **TiO_2_** | 459.10 | 7023.12 | 2.00 |
|  | 464.26 | 3000.84 | 1.96 |

**Table S5-2**. C 1s high-resolution XPS fitting parameters of Ti_3_C_2_T*_x_* (NH_4_F+thiourea).

| **Peak** | **Position** | **Area** | **FWHM (eV)** |
| --- | --- | --- | --- |
| **C-Ti** | 282.03 | 7159.19 | 0.66 |
| **C-C** | 284.21 | 1703.99 | 2.38 |
| **C-O** | 285.71 | 1542.26 | 2.17 |
| **C=O** | 288.20 | 515.29 | 1.75 |

**Table S5-3**. O 1s high-resolution XPS fitting parameters of Ti_3_C_2_T*_x_* (NH_4_F+thiourea).

| **Peak** | **Position** | **Area** | **FWHM (eV)** |
| --- | --- | --- | --- |
| **TiO_2_** | 529.91 | 8999.97 | 0.70 |
| **C-Ti-O** | 530.58 | 5706.77 | 0.88 |
| **C-Ti-OH** | 531.46 | 6261.88 | 1.09 |
| **C-O, C=O** | 532.48 | 5158.40 | 1.35 |

**Table S5-4**. N 1s high-resolution XPS fitting parameters of Ti_3_C_2_T*_x_* (NH_4_F+thiourea).

| **Peak** | **Position** | **Area** | **FWHM (eV)** |
| --- | --- | --- | --- |
| **Ti-N** | 397.21 | 491.85 | 1.20 |
| **N-1** | 398.43 | 142.93 | 0.70 |
| **N-Q** | 400.67 | 2291.46 | 2.28 |

**Table S5-5**. F 1s high-resolution XPS fitting parameters of Ti_3_C_2_T*_x_* (NH_4_F+thiourea).

| **Peak** | **Position** | **Area** | **FWHM (eV)** |
| --- | --- | --- | --- |
| **C-Ti-F** | 685.23 | 14793.92 | 1.23 |
| **AlF*_x_*** | 686.64 | 2463.37 | 1.37 |

**Table S5-6**. S 2p high-resolution XPS fitting parameters of Ti_3_C_2_T*_x_* (NH_4_F+thiourea).

| **Peak** | **Position** | **Area** | **FWHM (eV)** |
| --- | --- | --- | --- |
| **C=S** | 162.96 | 536.94 | 1.53 |
|  | 164.11 | 268.47 | 1.67 |

**Table S5**-**7.** The terminal groups’ ratio of Ti_3_C_2_T*_x_* MXene (NH_4_F+thiourea) calculated from their XPS spectra.

| **Terminal group** | **Area** | **SF** | **Atomic %** |
| --- | --- | --- | --- |
| **C-Ti-O** | 5706.77 | 2.88 | 23.37% |
| **C-Ti-OH** | 6261.88 | 2.88 | 25.65% |
| **Ti-N** | 491.85 | 1.67 | 3.47% |
| **C-Ti-F** | 14793.92 | 4.11 | 42.46% |
| **C=S** | 805.41 | 1.88 | 5.05% |

Calculation: The ratios of terminal groups were calculated from their normalized peak area. Further, the normalized peak area can be calculated from (Peak Area)/(SF), where SF is sensitivity factor.

Table S6-1. Mo 3d high-resolution XPS fitting parameters of Mo_2_TiC_2_T*_x_* (NH_4_F+thiourea).

| **Peak** | **Position** | **Area** | **FWHM (eV)** |
| --- | --- | --- | --- |
| **C-Mo-T*_x_*** | 228.86 | 7606.64 | 0.77 |
|  | 231.91 | 5071.91 | 0.53 |
| **C-Mo^4+^** | 229.28 | 30095.16 | 0.72 |
|  | 232.45 | 20063.44 | 0.66 |
| **C-Mo^5+^** | 229.60 | 15202.51 | 0.59 |
|  | 232.93 | 10135.00 | 0.71 |

**Table S6-2.** Ti 2p high-resolution XPS fitting parameters of Mo_2_TiC_2_T*_x_* (NH_4_F+thiourea).

| **Peak** | **Position** | **Area** | **FWHM (eV)** |
| --- | --- | --- | --- |
| **C-Ti^2+^** | 455.69 | 12902.87 | 1.11 |
|  | 461.67 | 6062.26 | 1.80 |
| **C-Ti^3+^** | 456.70 | 4068.98 | 1.46 |
|  | 462.63 | 2712.65 | 1.85 |

**Table S6-3.** C 1s high-resolution XPS fitting parameters of Mo_2_TiC_2_T*_x_* (NH_4_F+thiourea).

| **Peak** | **Position** | **Area** | **FWHM (eV)** |
| --- | --- | --- | --- |
| **C-Mo(Ti)** | 282.98 | 7328.53 | 0.77 |
| **C-C** | 284.64 | 8664.60 | 1.39 |
| **C-O** | 285.78 | 1098.58 | 1.55 |
| **C=O** | 288.58 | 414.41 | 0.94 |

**Table S6-4.** O 1s high-resolution XPS fitting parameters of Mo_2_TiC_2_T*_x_* (NH_4_F+thiourea).

| **Peak** | **Position** | **Area** | **FWHM (eV)** |
| --- | --- | --- | --- |
| **MoO*_x_*** | 530.07 | 7963.90 | 0.71 |
| **C-Mo-O** | 530.52 | 3831.33 | 0.78 |
| **C-Mo-OH** | 531.77 | 3072.16 | 1.30 |
| **C-O, C=O** | 532.94 | 545.37 | 0.53 |

**Table S6-5.** N 1s high-resolution XPS fitting parameters of Mo_2_TiC_2_T*_x_* (NH_4_F+thiourea).

| **Peak** | **Position** | **Area** | **FWHM (eV)** |
| --- | --- | --- | --- |
| **Mo-N** | 397.42 | 6632.64 | 1.64 |
| **N-2** | 399.7 | 5164.7 | 2.03 |

**Table S6-6.** F 1s high-resolution XPS fitting parameters of Mo_2_TiC_2_T*_x_* (NH_4_F+thiourea).

| **Peak** | **Position** | **Area** | **FWHM (eV)** |
| --- | --- | --- | --- |
| **C-Mo-F** | 685.01 | 903.55 | 1.68 |
| **AlF*_x_*** | 686.70 | 201.22 | 1.20 |

**Table S6-7.** S 2p high-resolution XPS fitting parameters of Mo_2_TiC_2_T*_x_* (NH_4_F+thiourea).

| **Peak** | **Position** | **Area** | **FWHM (eV)** |
| --- | --- | --- | --- |
| **C=S** | 162.95 | 1733.61 | 0.92 |
|  | 163.74 | 866.80 | 1.30 |
| **Mo-S** | 161.61 | 2173.11 | 0.76 |
|  | 162.28 | 1086.55 | 0.80 |

**Table S6-8.** The terminal groups’ ratio of Mo_2_TiC_2_T*_x_* MXene (NH_4_F+thiourea) calculated from their XPS spectra.

| **Terminal group** | **Area** | **SF** | **Atomic %** |
| --- | --- | --- | --- |
| **C-Mo-O** | 3831.33 | 2.88 | 15.98% |
| **C-Mo-OH** | 3072.16 | 2.88 | 12.82% |
| **Mo-N** | 6632.64 | 1.67 | 47.72% |
| **C-Mo-F** | 903.55 | 4.11 | 2.64% |
| **Mo-S** | 3259.66 | 1.88 | 20.84% |

Table S7-1. V 2p high-resolution XPS fitting parameters of V_4_C_3_T*_x_* (NH_4_F+thiourea).

| **Peak** | **Position** | **Area** | **FWHM (eV)** |
| --- | --- | --- | --- |
| **C-V-T*_x_*** | 513.43 | 18701.08 | 0.63 |
|  | 520.72 | 7532.58 | 1.30 |
| **C-V^3+^** | 514.08 | 14652.30 | 0.61 |
|  | 521.48 | 6198.75 | 1.11 |
| **C-V^4+^** | 515.30 | 13758.32 | 1.83 |
|  | 522.63 | 6879.16 | 3.14 |

**Table S7-2.** C 1s high-resolution XPS fitting parameters of V_4_C_3_T*_x_* (NH_4_F+thiourea).

| **Peak** | **Position** | **Area** | **FWHM (eV)** |
| --- | --- | --- | --- |
| **C-V** | 282.77 | 5447.74 | 0.61 |
| **C-C** | 284.43 | 7968.40 | 1.33 |
| **C-O** | 285.36 | 2015.00 | 1.20 |
| **C=O** | 287.51 | 400.58 | 1.75 |

**Table S7-3.** N 1s high-resolution XPS fitting parameters of V_4_C_3_T*_x_* (NH_4_F+thiourea).

| **Peak** | **Position** | **Area** | **FWHM (eV)** |
| --- | --- | --- | --- |
| **V-N** | 397.24 | 376.64 | 0.95 |
| **N-2** | 399.32 | 1131.59 | 2.21 |
| **N-Q** | 401.05 | 391.14 | 2.14 |

**Table S7-4.** O 1s high-resolution XPS fitting parameters of V_4_C_3_T*_x_* (NH_4_F+thiourea).

| **Peak** | **Position** | **Area** | **FWHM (eV)** |
| --- | --- | --- | --- |
| **VO*_x_*** | 529.53 | 1525.17 | 0.76 |
| **C-V-O** | 530.06 | 606.15 | 0.72 |
| **C-V-OH** | 531.05 | 1098.27 | 1.30 |
| **C-O, C=O** | 531.98 | 2734.2 | 1.71 |

**Table S7-5.** F 1s high-resolution XPS fitting parameters of V_4_C_3_T*_x_* (NH_4_F+thiourea).

| **Peak** | **Position** | **Area** | **FWHM (eV)** |
| --- | --- | --- | --- |
| **C-V-F** | 684.06 | 1421.54 | 1.86 |
| **AlF*_x_*** | 685.85 | 1264.79 | 2.01 |

**Table S7-6.** S 2p high-resolution XPS fitting parameters of V_4_C_3_T*_x_* (NH_4_F+thiourea).

| **Peak** | **Position** | **Area** | **FWHM (eV)** |
| --- | --- | --- | --- |
| **C=S** | 162.57 | 1346.77 | 0.96 |
|  | 163.48 | 673.38 | 1.24 |
| **V-S** | 161.18 | 3271.43 | 0.62 |
|  | 162.11 | 1264.25 | 0.69 |

**Table S7-7.** The terminal groups’ ratio of V_4_C_3_T*_x_* MXene (NH_4_F+thiourea) calculated from their XPS spectra.

| **Terminal group** | **Area** | **SF** | **Atomic %** |
| --- | --- | --- | --- |
| **C-V-O** | 606.15 | 2.88 | 5.89% |
| **C-V-OH** | 1098.27 | 2.88 | 10.66% |
| **V-N** | 376.64 | 1.67 | 6.31% |
| **C-V-F** | 1421.54 | 4.11 | 9.67% |
| **V-S** | 4535.68 | 1.88 | 67.47% |

Table S8-1. V 2p high-resolution XPS fitting parameters of V_2_CT*_x_* (NH_4_F+thiourea).

| **Peak** | **Position** | **Area** | **FWHM (eV)** |
| --- | --- | --- | --- |
| **C-V-T*_x_*** | 513.31 | 8867.65 | 1.04 |
|  | 520.82 | 3846.49 | 2.01 |
| **C-V^3+^** | 514.39 | 7027.80 | 1.62 |
|  | 521.65 | 3297.71 | 2.64 |
| **C-V^4+^** | 516.49 | 7098.46 | 2.39 |
|  | 523.47 | 2307.37 | 2.80 |

**Table S8-2.** C 1s high-resolution XPS fitting parameters of V_2_CT*_x_* (NH_4_F+thiourea).

| **Peak** | **Position** | **Area** | **FWHM (eV)** |
| --- | --- | --- | --- |
| **C-V** | 282.55 | 990.47 | 0.58 |
| **C-C** | 284.23 | 6278.13 | 1.63 |
| **C-O** | 285.15 | 2065.32 | 1.45 |
| **C=O** | 287.60 | 400.00 | 2.50 |

**Table S8-3.** N 1s high-resolution XPS fitting parameters of V_2_CT*_x_* (NH_4_F+thiourea).

| **Peak** | **Position** | **Area** | **FWHM (eV)** |
| --- | --- | --- | --- |
| **V-N** | 397.67 | 200.32 | 1.60 |
| **N-2** | 399.52 | 1508.49 | 1.69 |
| **N-Q** | 401.197 | 1988.16 | 1.68 |

**Table S8-4.** O 1s high-resolution XPS fitting parameters of V_2_CT*_x_* (NH_4_F+thiourea).

| **Peak** | **Position** | **Area** | **FWHM (eV)** |
| --- | --- | --- | --- |
| **VO*_x_*** | 529.60 | 997.45 | 1.17 |
| **C-V-O** | 530.53 | 2026.62 | 1.14 |
| **C-V-OH** | 531.37 | 2773.2 | 1.28 |
| **C-O, C=O** | 532.28 | 2210.58 | 1.63 |

**Table S8-5.** F 1s high-resolution XPS fitting parameters of V_2_CT*_x_* (NH_4_F+thiourea).

| **Peak** | **Position** | **Area** | **FWHM (eV)** |
| --- | --- | --- | --- |
| **C-V-F** | 684.56 | 17443.46 | 1.91 |
| **AlF*_x_*** | 685.69 | 1303.85 | 1.20 |

**Table S8-6.** S 2p high-resolution XPS fitting parameters of V_2_CT*_x_* (NH_4_F+thiourea).

| **Peak** | **Position** | **Area** | **FWHM (eV)** |
| --- | --- | --- | --- |
| **C=S** | 162.12 | 2255.27 | 2.01 |
|  | 164.00 | 1127.63 | 2.50 |
| **V-S** | 160.87 | 3102.94 | 0.65 |
|  | 162.02 | 1551.47 | 0.75 |

**Table S8-7.** The terminal groups’ ratio of V_2_CT*_x_* MXene (NH_4_F+thiourea) calculated from their XPS spectra.

| **Terminal group** | **Area** | **SF** | **Atomic %** |
| --- | --- | --- | --- |
| **C-V-O** | 2026.62 | 2.88 | 8.27% |
| **C-V-OH** | 2773.20 | 2.88 | 11.32% |
| **V-N** | 200.00 | 1.67 | 1.41% |
| **C-V-F** | 17443.46 | 4.11 | 49.89% |
| **V-S** | 4654.41 | 1.88 | 29.11% |

Table S9-1. Nb 3d high-resolution XPS fitting parameters of Nb_2_CT*_x_* (NH_4_F+thiourea).

| **Peak** | **Position** | **Area** | **FWHM (eV)** |
| --- | --- | --- | --- |
| **C-Nb-T*_x_*** | 204.04 | 6807.04 | 0.58 |
|  | 206.76 | 4538.03 | 0.69 |
| **C-Nb^4+^** | 204.69 | 4538.03 | 0.94 |
|  | 208.42 | 3025.35 | 0.55 |
| **C-Nb^5+^** | 207.83 | 27630.16 | 1.24 |
|  | 210.71 | 13378.05 | 0.98 |

**Table S9-2.** C 1s high-resolution XPS fitting parameters of Nb_2_CT*_x_* (NH_4_F+thiourea).

| **Peak** | **Position** | **Area** | **FWHM (eV)** |
| --- | --- | --- | --- |
| **C-Nb** | 282.70 | 631.72 | 0.69 |
| **C-C** | 284.80 | 3641.28 | 1.30 |
| **C-O** | 285.73 | 2079.35 | 1.51 |
| **C=O** | 287.31 | 1114.32 | 2.52 |

**Table S9-3.** N 1s high-resolution XPS fitting parameters of Nb_2_CT*_x_* (NH_4_F+thiourea).

| **Peak** | **Position** | **Area** | **FWHM (eV)** |
| --- | --- | --- | --- |
| **Nb-N** | 397.08 | 1080.94 | 1.74 |
| **N-2** | 400.15 | 2298.75 | 3.54 |
| **N-Q** | 401.39 | 700.00 | 3.43 |

**Table S9-4.** O 1s high-resolution XPS fitting parameters of Nb_2_CT*_x_* (NH_4_F+thiourea).

| **Peak** | **Position** | **Area** | **FWHM (eV)** |
| --- | --- | --- | --- |
| **NbO*_x_*** | 530.35 | 3629.88 | 0.70 |
| **C-Nb-O** | 530.89 | 7498.38 | 0.67 |
| **C-Nb-OH** | 531.41 | 7214.57 | 0.77 |
| **C-O, C=O** | 532.25 | 3587.41 | 1.13 |

**Table S9-5.** F 1s high-resolution XPS fitting parameters of Nb_2_CT*_x_* (NH_4_F+thiourea).

| **Peak** | **Position** | **Area** | **FWHM (eV)** |
| --- | --- | --- | --- |
| **C-Nb-F** | 685.02 | 6872.78 | 1.39 |
| **AlF*_x_*** | 686.16 | 283.36 | 0.65 |

**Table S9-6.** S 2p high-resolution XPS fitting parameters of Nb_2_CT*_x_* (NH_4_F+thiourea).

| **Peak** | **Position** | **Area** | **FWHM (eV)** |
| --- | --- | --- | --- |
| **C=S** | 162.21 | 515.75 | 0.77 |
|  | 163.82 | 257.87 | 1.19 |
| **Nb-S** | 161.08 | 844.40 | 0.62 |
|  | 162.54 | 422.21 | 1.41 |

**Table S9-7.** The terminal groups’ ratio of Nb_2_CT*_x_* MXene (NH_4_F+thiourea) calculated from their XPS spectra.

| **Terminal group** | **Area** | **SF** | **Atomic %** |
| --- | --- | --- | --- |
| **C-Nb-O** | 7498.38 | 2.88 | 32.14% |
| **C-Nb-OH** | 7214.57 | 2.88 | 30.92% |
| **Nb-N** | 1080.94 | 1.67 | 7.99% |
| **C-Nb-F** | 6872.78 | 4.11 | 20.64% |
| **Nb-S** | 1266.61 | 1.88 | 8.31% |

Table S10-1. Nb 3d high-resolution XPS fitting parameters of Nb_4_C_3_T*_x_* (NH_4_F+thiourea).

| **Peak** | **Position** | **Area** | **FWHM (eV)** |
| --- | --- | --- | --- |
| **C-Nb-T*_x_*** | 204.06 | 7472.74 | 0.61 |
|  | 206.79 | 4981.83 | 0.66 |
| **C-Nb^4+^** | 204.79 | 4981.83 | 1.44 |
|  | 208.45 | 3321.22 | 0.55 |
| **C-Nb^5+^** | 207.82 | 23658.15 | 1.08 |
|  | 210.69 | 15772.10 | 1.10 |

**Table S10-2.** C 1s high-resolution XPS fitting parameters of Nb_4_C_3_T*_x_* (NH_4_F+thiourea).

| **Peak** | **Position** | **Area** | **FWHM (eV)** |
| --- | --- | --- | --- |
| **C-Nb** | 282.65 | 2849.36 | 0.90 |
| **C-C** | 284.70 | 3964.91 | 1.29 |
| **C-O** | 285.53 | 2203.41 | 1.75 |
| **C=O** | 288.35 | 895.10 | 2.80 |

**Table S10-3.** N 1s high-resolution XPS fitting parameters of Nb_4_C_3_T*_x_* (NH_4_F+thiourea).

| **Peak** | **Position** | **Area** | **FWHM (eV)** |
| --- | --- | --- | --- |
| **Nb-N** | 397.11 | 314.73 | 1.02 |
| **N-2** | 399.80 | 2004.41 | 2.37 |
| **N-Q** | 401.70 | 1274.93 | 3.10 |

**Table S10-4.** O 1s high-resolution XPS fitting parameters of Nb_4_C_3_T*_x_* (NH_4_F+thiourea).

| **Peak** | **Position** | **Area** | **FWHM (eV)** |
| --- | --- | --- | --- |
| **NbO*_x_*** | 529.95 | 1880.87 | 0.89 |
| **C-Nb-O** | 530.70 | 2779.57 | 0.99 |
| **C-Nb-OH** | 531.44 | 2245.62 | 1.08 |
| **C-O, C=O** | 532.42 | 2256.82 | 1.40 |

**Table S10-5.** F 1s high-resolution XPS fitting parameters of Nb_4_C_3_T*_x_* (NH_4_F+thiourea).

| **Peak** | **Position** | **Area** | **FWHM (eV)** |
| --- | --- | --- | --- |
| **C-Nb-F** | 684.37 | 5111.74 | 1.79 |
| **AlF*_x_*** | 686.93 | 664.81 | 3.83 |

**Table S10-6.** S 2p high-resolution XPS fitting parameters of Nb_4_C_3_T*_x_* (NH_4_F+thiourea).

| **Peak** | **Position** | **Area** | **FWHM (eV)** |
| --- | --- | --- | --- |
| **C=S** | 162.53 | 668.40 | 1.74 |
|  | 163.98 | 334.20 | 1.55 |
| **Nb-S** | 161.00 | 1077.05 | 0.91 |
|  | 162.13 | 538.53 | 0.97 |

**Table S10-7.** The terminal groups’ ratio of Nb_4_C_3_T*_x_* MXene (NH_4_F+thiourea) calculated from their XPS spectra.

| **Terminal group** | **Area** | **SF** | **Atomic %** |
| --- | --- | --- | --- |
| **C-Nb-O** | 2779.57 | 2.88 | 23.91% |
| **C-Nb-OH** | 2245.62 | 2.88 | 19.32% |
| **Nb-N** | 314.73 | 1.67 | 4.67% |
| **C-Nb-F** | 5111.74 | 4.11 | 30.81% |
| **Nb-S** | 1615.58 | 1.88 | 21.29% |

**Table S11.** Comparison of synthesis parameters for preparing MXenes by the anhydrous NH_4_F+thiourea method and conventional etching methods.

| **MXene** | **Methods** | **Etchant** | **Temperature** | **Time (h)** | **Yield(%)** | **Production rate (mg/min)** | **Experimental equipment** | **Ref** |
| --- | --- | --- | --- | --- | --- | --- | --- | --- |
| Ti_3_C_2_T*_x_* | Hydrothermal synthesis | 4 M NH_4_F | 200°C | 6 | - | < 0.55 | Autoclave | ^9^ |
| V_2_CT*_x_* | Hydrothermal synthesis | 4 M NH_4_F | 220°C | 36 | - | < 0.14 | Autoclave | ^9^ |
| Nb_4_C_3_T*_x_* | Hydrothermal synthesis | 8 M NH_4_F | 200°C | 6 | -- | < 0.55 | Autoclave | ^9^ |
| V_2_CT*_x_* | Gas-solid etching | PVDF | 600°C | 0.5 | -- | -- | Atmosphere furnace | ^10^ |
| Ti_3_C_2_T*_x_* | Gas-solid etching | PVDF | 600°C | 0.5 | -- | -- | Atmosphere furnace | ^10^ |
| Nb_2_CT*_x_* | Gas-solid etching | PVDF | 550°C | 0.5 | -- | -- | Atmosphere furnace | ^10^ |
| Ti_3_C_2_T*_x_* | Organic etching | CF_3_SO_3_H | RT | 6.5 | 70 | < 1.38 | -- | ^11^ |
| Ti_3_C_2_T*_x_* | Organic etching | NH_4_HF_2_ + PC | 35°C | 196 | -- | < 0.09 | Ar-filled glovebox | ^12^ |
| Ti_3_C_2_T*_x_* | Gas-solid etching | FeCl_3_ | 600°C-700°C | 12-24 | -- | -- | Atmosphere furnace | ^13^ |
| Ti_3_C_2_T*_x_* | Molten-salt etching | CdBr_2_ | 650°C | 12 | -- | < 0.14 | Atmosphere furnace | ^14^ |
| Ti_3_C_2_T*_x_* | MILD | HCl+LiF | RT | 24 | -- | < 0.58 | -- | ^15^ |
| Ti_3_C_2_T*_x_* | HF etching | 48-51% HF | 50°C | 48 | -- | < 0.70 | -- | ^15^ |
| Nb_2_CT*_x_* | Molten-salt etching | AgCl | 700°C | -- | -- | -- | Atmosphere furnace | ^16^ |
| Nb_2_CT*_x_* | Molten-salt etching | CdCl_2_ | 710°C | 36 |  | < 0.26 | Atmosphere furnace | ^17^ |
| Ti_3_C_2_T*_x_* | Electrochemical etching | NaBF_4_/HCl/ DMSO | RT | 48 | 60 | < 0.52 | Electrochemical equipment | ^18^ |
| Ti_3_C_2_T*_x_* | Electrochemical etching | LiOH/LiCl | RT | 5 | 92.2 | -- | Electrochemical equipment | ^19^ |
| Ti_3_C_2_T*_x_* | Photo-Fenton etching | Fenton’s solution | -- | 10 | -- | < 0.50 | UV lamp | ^20^ |
| V_2_CT*_x_* | Electrochemical etching | 21 M LiTFSI + 1 M Zn(OTf)_2_ | RT | -- | -- | -- | Electrochemical equipment | ^21^ |
| V_2_CT*_x_* | HF etching | 24%HF | 30°C | 56 | -- | < 0.15 | -- | ^22^ |
| Nb_2_CT*_x_* | Electrochemical etching | 0.5 M HCl | 50°C | 4 | 50 | 0.02 | Electrochemical equipment | ^23^ |
| Ti_3_C_2_T*_x_* | Electrochemical etching | NH_4_Cl+TMAOH | RT | 5 | 90 | -- | Electrochemical equipment | ^24^ |
| Ti_3_C_2_T*_x_* | Electrochemical etching | 2-18 M NH_4_F | RT | -- | 67.4-71.2 | 0.03-2.13 | Electrochemical equipment | ^25^ |
| Nb_2_CT*_x_* | Molten-salt etching | CuCl_2_ | 750°C | 24 | -- | -- | Atmosphere furnace | ^26^ |
| Mo_2_TiC_2_T_x_ | HF etching | 45%HF | 55°C | 48 | -- | -- | -- | ^27^ |
| V_4_C_3_T_x_ | -- | Te | 700°C | 1 | -- | -- | Atmosphere furnace | ^28^ |
| Nb_4_C_3_T*_x_* | HF etching | 49%HF | RT | 140 | -- | < 0.05 | -- | ^29^ |
| V_4_C_3_T*_x_* | HF etching | 49%HF | 55°C | 120 | < 50 | < 0.12 | -- | ^30^ |
| Mo_2_TiC_2_T_x_ | HF etching | 49%HF | RT | 144 | -- | < 0.12 | -- | ^31^ |
| Mo_2_TiC_2_T_x_ | HF etching | 49%HF | 55°C | 48 | -- | < 0.28 | -- | ^32^ |
| **Ti_3_C_2_T*_x_*** | **Molten-salt etching** | **NH_4_F+thiourea** | **200°C** | **2** | **86.7** | **1.81** | **Autoclave** | **This work** |
| **Mo_2_TiC_2_T*_x_*** | **Molten-salt etching** | **NH_4_F+thiourea** | **200°C** | **24** | **92.3** | **0.16** | **Autoclave** | **This work** |
| **Ti_3_C_2_T*_x_*** | **Molten-salt etching** | **NH_4_F+thiourea** | **200°C** | **2** | **86.7** | **1.81** | **Autoclave** | **This work** |
| **V_2_CT_x_** | **Molten-salt etching** | **NH_4_F+thiourea** | **200°C** | **6** | **73.2** | **0.51** | **Autoclave** | **This work** |
| **V_4_C_3_T_x_** | **Molten-salt etching** | **NH_4_F+thiourea** | **200°C** | **24** | **94.3** | **0.16** | **Autoclave** | **This work** |
| **Nb_2_CT_x_** | **Molten-salt etching** | **NH_4_F+thiourea** | **200°C** | **6** | **80.1** | **0.56** | **Autoclave** | **This work** |
| **Nb_4_C_3_T*_x_*** | **Molten-salt etching** | **NH_4_F+thiourea** | **200°C** | **24** | **89.3** | **0.16** | **Autoclave** | **This work** |

**Table S12.** Comparison of etchant prices for the NH_4_F + thiourea method and other etching methods.

| **Etchant** | **Packaging specifications** | **Price** | **Unit-price** | **Supplier** |
| --- | --- | --- | --- | --- |
| HF (40%) | 1000 mL | CNY 630 | CNY 0.63/mL | Aladdin |
| LiF | 1000 g | CNY 3660 | CNY 3.66/g | Aladdin |
| PVDF | 500 g | CNY 620 | CNY 1.24/g | Aladdin |
| CF_3_SO_3_H | 10 g | CNY 130 | CNY 1.30/g | Aladdin |
| NH_4_HF_2_ | 500 g | CNY 76 | CNY 0.15/g | Aladdin |
| FeCl_3_ | 100 g | CNY 210 | CNY 2.10/g | Aladdin |
| CdBr_2_ | 25 g | CNY 650 | CNY 24.80/g | Aladdin |
| LiF | 500 g | CNY 814 | CNY 1.63/g | Aladdin |
| HCl (36%) | 500 mL | CNY 31 | CNY 0.06/mL | Sinopharm |
| AgCl | 1 g | CNY 315 | CNY 315.00/g | Aladdin |
| NaBF_4_ | 500 g | CNY 376 | CNY 0.75/g | Aladdin |
| LiOH | 100 g | CNY 258 | CNY 2.58/g | Aladdin |
| LiCl | 100 g | CNY 155 | CNY 1.55/g | Aladdin |
| LiTFSI | 10 g | CNY 80 | CNY 8.00/g | Aladdin |
| Zn(OTf)_2_ | 1 g | CNY 230 | CNY 230.00/g | Aladdin |
| NH_4_Cl | 500 g | CNY 410 | CNY 0.82/g | Aladdin |
| CuCl_2_ | 100 g | CNY 50 | CNY 0.50/g | Aladdin |
| CoCl_2_ | 100 g | CNY 1491 | CNY 14.91/g | Sinopharm |
| SnCl_2_ | 250 g | CNY 229 | CNY 0.91/g | Sinopharm |
| **NH_4_F** | **500 g** | **CNY 89** | **CNY 0.18/g** | **Aladdin** |

**Table S13.** Comparison of the small-molecule intercalants in the present work and other reported intercalated species.

| **Intercalant** | **Packaging specifications** | **Price** | **Unit-price** | **Supplier** |
| --- | --- | --- | --- | --- |
| TBAOH (25%) | 50 mL | CNY 91 | CNY 1.82/mL | Aladdin |
| TBAOH (55%) | 100 mL | CNY 600 | CNY 6.00/mL | Aladdin |
| TMAOH (25%) | 100 mL | CNY 70 | CNY 0.70/mL | Aladdin |
| TPAOH (1 M) | 50 g | CNY 1272 | CNY 2.54/g | Aladdin |
| TPAOH (40%) | 100 g | CNY 40 | CNY 0.40/g | Aladdin |
| DMSO | 1000 mL | CNY 304 | CNY 0.30/mL | Aladdin |
| LiCl | 100 g | CNY 155 | CNY 1.55/g | Aladdin |
| **Urea** | **500 g** | **CNY 169** | **CNY 0.34/g** | **Aladdin** |
| **Thiourea** | **500 g** | **CNY 80** | **CNY 0.16/g** | **Aladdin** |
| **Oxalic acid** | **100 g** | **CNY 46** | **CNY 0.46/g** | **Aladdin** |
| **Acetamide** | **250 g** | **CNY 54** | **CNY 0.22/g** | **Aladdin** |
| **Propionamide** | **100 g** | **CNY 70** | **CNY 0.70g** | **Aladdin** |
| **Glycine** | **500 g** | **CNY 81** | **CNY 0.16/g** | **Aladdin** |

Table S14. Summary of the HER performance of three MXenes in 0.5 M H_2_SO_4_.

| **Samples** | **Mass loading (mg cm^-2^)** | **η_10_**  **(mV *vs.* RHE)** | **Tafel slope**  **(mV dec^-1^)** | **C_dl_**  **(mF cm^-2^)** |
| --- | --- | --- | --- | --- |
| Ti_3_C_2_T*_x_* | 0.2 | 320.0 | 90.4 | 0.4 |
| Mo_2_TiC_2_T*_x_* | 0.2 | 251.6 | 80.6 | 9.7 |
| V_4_C_3_T*_x_* | 0.2 | 304.6 | 82.3 | 20.2 |

**Table S15.** The fitted parameters of the EIS data of three MXenes for HER in 0.5 M H_2_SO_4_.

| Catalysts | R_s_ (Ω) | R_ct_ (Ω) | CPE-T | CPE-P |
| --- | --- | --- | --- | --- |
| Ti_3_C_2_T*_x_* | 10.73 | 53.88 | 1.66E-5 | 0.80 |
| Mo_2_TiC_2_T*_x_* | 10.98 | 14.59 | 1.22E-3 | 0.83 |
| V_4_C_3_T*_x_* | 11.72 | 30.90 | 1.87E-2 | 0.59 |

**Table S16.** Summary of HER performance of various MXenes and some non-precious metal materials.

| **Catalyst** | **Electrolyte** | **η_10_ (mV)** | **Tafel slope (mV dec^-1^)** | **Synthetic time** | **Synthetic strategy** |
| --- | --- | --- | --- | --- | --- |
| **Mo_2_TiC_2_T_x_** | 0.5 M H_2_SO_4_ | 251 | 80 | 24 h | This work |
| **Ti_3_C_2_T_x_** | 0.5 M H_2_SO_4_ | > 320 | 90 | 2 h | This work |
| **V_4_C_3_T_x_** | 0.5 M H_2_SO_4_ | 304 | 82 | 24 h | This work |
| **Ti_3_C_2_T_x_** | 0.5 M H_2_SO_4_ | 843 | 340 | 24 h | 9 M HCl+LiF ^33^ |
| **Ti_2_CT_x_** | 0.5 M H_2_SO_4_ | 609 | 124 | 18 h | 10%HF ^34^ |
| **Nb_2_CT_x_** | 0.5 M H_2_SO_4_ | > 300 | 239 | -- | purchased ^35^ |
| **Nb_4_C_3_T_x_** | 0.5 M H_2_SO_4_ | 398 | 122 | 180 h | 49%HF ^36^ |
| **Mo_2_CT_x_** | 0.5 M H_2_SO_4_ | 283 | 82 | 7 d | 50%HF ^34^ |
| **V_4_C_3_T_x_** | 0.5 M H_2_SO_4_ | ~800 | 236 | 165 h | 40%HF ^37^ |
| **V_2_CT_x_** | 0.5 M H_2_SO_4_ | 760 | 187 | 100 h | 45%-49% HF ^38^ |
| **Mo_2_TiC_2_T_x_** | 0.5 M H_2_SO_4_ | 248 | 74 | 48 h | 50%HF ^39^ |
| **Mo_2_Ti_2_C_3_T_x_** | 0.5 M H_2_SO_4_ | 275 | 99 | 90 h | 50%HF ^39^ |
| **Mo_1.33_C** | 0.1 M HClO_4_ | 422 | -- | 24 h | 28M HF ^40^ |
| **W_1.33_C** | 0.1 M HClO_4_ | 320 | -- | 30 h | 48%HF^41^ |

**References**

1	Chen, Y. *et al.* First‐principles study on the structural, phonon, and thermodynamic properties of the ternary carbides in Ti–Al–C system. *Physica Status Solidi (A) - Applications and Materials Science* **208**, 1879-1884, doi:10.1002/pssa.201127020 (2011).

2	Ma, S., Jiao, Z. & Huang, X. First-principles study of ceramic material (Ti_1−x_Nb_x_)_2_AlC compounds and its compressive behavior under pressure up to 55 GPa. *Journal of Alloys and Compounds* **591**, 110-116, doi:10.1016/j.jallcom.2013.12.233 (2014).

3	Hu, C. *et al.* Nb_4_AlC_3_: A new compound belonging to the MAX phases. *Scripta Materialia* **57**, 893-896, doi:10.1016/j.scriptamat.2007.07.038 (2007).

4	J.C. Schuster, H. Nowotny & Vaccaro, C. The ternary systems: Cr-Al-C, V-Al-C, and Ti-Al-C and the behavior of H-phases (M_2_AlC). *Journal of Solid State Chemistry* **32**, 213–219 (1980).

5	Du, Y. L., Sun, Z. M., Hashimoto, H. & Tian, W. B. Bonding properties and bulk modulus of M_4_AlC_3_ (M = V, Nb, and Ta) studied by first‐principles calculations. *Physica Status Solidi (A) - Applications and Materials Science* **246**, 1039-1043, doi:10.1002/pssb.200844449 (2009).

6	Yang, C. *et al.* Flexible nitrogen-doped 2D titanium carbides (MXene) films constructed by an ex situ solvothermal method with extraordinary volumetric capacitance. *Advanced Energy Materials* **8**, 1802087, doi:10.1002/aenm.201802087 (2018).

7	Wen, Y. *et al.* Nitrogen-doped Ti_3_C_2_T_x_ MXene electrodes for high-performance supercapacitors. *Nano Energy* **38**, 368-376, doi:<http://dx.doi.org/10.1016/j.nanoen.2017.06.009> (2017).

8	Wei, H. *et al.* Incorporating ultra-small N-doped Mo_2_C nanoparticles onto 3D N-doped flower-like carbon nanospheres for robust electrocatalytic hydrogen evolution. *Nano Energy* **86**, 106047, doi:10.1016/j.nanoen.2021.106047 (2021).

9	Xu, H. *et al.* Halogen Ion-Mediated Hydrothermal Synthesis of Diverse MXenes with Tailored Heterostructures. *Advanced Materials* **37**, e2504586, doi:10.1002/adma.202504586 (2025).

10	Liu, Y. *et al.* General and fast gas-solid synthesis of functional MXenes and derivatives on the scale of tens of grams. *Angewandte Chemie International Edition* **26**, e202420287, doi:10.1002/anie.202420287 (2025).

11	Wang, F. *et al.* One-step synthesis of organic terminal 2D Ti_3_C_2_T_x_ MXene nanosheets by etching of Ti_3_AlC_2_ in an organic lewis acid solvent. *Angewandte Chemie International Edition* **63**, e202405315, doi:10.1002/anie.202405315 (2024).

12	Natu, V. *et al.* 2D Ti_3_C_2_T_z_ MXene synthesized by water-free etching of Ti_3_AlC_2_ in polar organic solvents. *Chem* **6**, 616-630, doi:10.1016/j.chempr.2020.01.019 (2020).

13	Zhu, J. *et al.* Solvent-free one-step green synthesis of MXenes by “gas-phase selective etching”. *Energy Storage Materials* **70**, 103503, doi:10.1016/j.ensm.2024.103503 (2024).

14	Fan, Q. *et al.* Gaseous molecules-mediated electrochemical exfoliation of halogenated MXenes and its boosting in wear-resisting tribovoltaic devices. *Nature Communications* **16**, 5051, doi:10.1038/s41467-025-60303-5 (2025).

15	Hart, J. L. *et al.* Control of MXenes’ electronic properties through termination and intercalation. *Nature Communications* **10**, 522, doi:10.1038/s41467-018-08169-8 (2019).

16	Li, Y. *et al.* A general Lewis acidic etching route for preparing MXenes with enhanced electrochemical performance in non-aqueous electrolyte. *Nature Materials* **19**, 894-899, doi:10.1038/s41563-020-0657-0 (2020).

17	Vladislav Kamysbayev *et al.* Covalent surface modifications and superconductivity of two-dimensional metal carbide MXenes. *Science* **369**, 979-983 (2020).

18	Ostermann, M. *et al.* Pulsed electrochemical exfoliation for an HF-free sustainable mxene synthesis. *Small* **21**, e2500807, doi:10.1002/smll.202500807 (2025).

19	Chen, J. *et al.* Simplified synthesis of fluoride-free Ti_3_C_2_T_x_ via electrochemical etching toward high-performance electrochemical capacitors. *ACS Nano* **16**, 2461-2470, doi:10.1021/acsnano.1c09004 (2022).

20	Liang, L., Niu, L., Wu, T., Zhou, D. & Xiao, Z. Fluorine-free fabrication of MXene via photo-fenton approach for advanced lithium-sulfur batteries. *ACS Nano* **16**, 7971−7981, doi:10.1021/acsnano.2c00779 (2022).

21	Li, X. *et al.* In situ electrochemical synthesis of MXenes without acid/alkali usage in/for an aqueous zinc ion battery. *Advanced Energy Materials* **10**, 2001791, doi:10.1002/aenm.202001791 (2020).

22	Liu, Y. *et al.* In‐situ electrochemically activated surface vanadium valence in V_2_C MXene to achieve high capacity and superior rate performance for Zn‐ion batteries. *Advanced Functional Materials* **31**, 2203805, doi:10.1002/adfm.202008033 (2021).

23	Song, M., Pang, S. Y., Guo, F., Wong, M. C. & Hao, J. Fluoride-free 2D niobium carbide mxenes as stable and biocompatible nanoplatforms for electrochemical biosensors with ultrahigh sensitivity. *Advanced Science* **7**, 2001546, doi:10.1002/advs.202001546 (2020).

24	Yang, S. *et al.* Fluoride-free synthesis of two-dimensional titanium carbide (MXene) using a binary aqueous system. *Angewandte Chemie International Edition* **57**, 15491-15495, doi: 10.1002/anie.201809662 (2018).

25	Huang, Z. *et al.* Green and scalable electrochemical routes for cost‐effective mass production of MXenes for supercapacitor electrodes. *Carbon Energy* **5**, e295, doi:10.1002/cey2.295 (2023).

26	Li, D. *et al.* MXenes with ordered triatomic-layer borate polyanion terminations. *Nature Materials* **23**, 1085-1092, doi:10.1038/s41563-024-01911-2 (2024).

27	Zhang, J. *et al.* Single platinum atoms immobilized on an MXene as an efficient catalyst for the hydrogen evolution reaction. *Nature Catalysis* **1**, 985-992, doi:10.1038/s41929-018-0195-1 (2018).

28	Ma, G. *et al.* Tellurium-Terminated MXene Synthesis via One-Step Tellurium Etching. *Nano-Micro Letters* **18**, 28, doi:10.1007/s40820-025-01875-1 (2026).

29	Zhao, S. *et al.* Flexible Nb_4_C_3_T_x_ Film with Large Interlayer Spacing for High‐Performance Supercapacitors. *Advanced Functional Materials* **30**, 2000815, doi:10.1002/adfm.202000815 (2020).

30	Bin, X. *et al.* The synthesis and supercapacitor application of flexible free-standing Ti_3_C_2_T_x_, Mo_2_TiC_2_T_x_, and V_4_C_3_T_x_ MXene Films. *Nanoscale* **16**, 15196-15207, doi:10.1039/d4nr01826e (2024).

31	Luxa, J. *et al.* Hydrogen evolution reaction activity in Mo_2_TiC_2_T_x_ MXene derived from Mo_2_TiAlC_2_ MAX phase: Insights from compositional transformations. *ACS Catalysis* **14**, 15336-15347, doi:10.1021/acscatal.4c04099 (2024).

32	Wu, Z. *et al.* Mo_2_TiC_2_ MXene-supported Ru clusters for efficient photothermal reverse water-gas shift. *ACS Nano* **17**, 1550-1559, doi:10.1021/acsnano.2c10707 (2022).

33	Bat-Erdene, M. *et al.* Highly dispersed Ru nanoparticles on boron-doped Ti_3_C_2_T_x_ (MXene) nanosheets for synergistic enhancement of electrocatalytic hydrogen evolution. *Small* **17**, e2102218, doi:10.1002/smll.202102218 (2021).

34	Seh, Z. W. *et al.* Two-dimensional molybdenum carbide (MXene) as an efficient electrocatalyst for hydrogen evolution. *ACS Energy Letters* **1**, 589-594, doi:10.1021/acsenergylett.6b00247 (2016).

35	Fan, X. *et al.* Mechanochemical synthesis of Pt/Nb_2_CT_x_ MXene composites for enhanced electrocatalytic hydrogen evolution. *Materials* **14**, 2426, doi:10.3390/ma14092426 (2021).

36	Tan, Y. *et al.* Nb_4_C_3_T_x_ (MXene) as a new stable catalyst for the hydrogen evolution reaction. *International Journal of Hydrogen Energy* **46**, 1955-1966, doi:10.1016/j.ijhydene.2020.10.046 (2021).

37	Tran, M. H. *et al.* Adding a new member to the MXene family: Synthesis, structure, and electrocatalytic activity for the hydrogen evolution reaction of V_4_C_3_T_x_. *ACS Applied Energy Materials* **1**, 3908-3914, doi:10.1021/acsaem.8b00652 (2018).

38	Yoon, Y. *et al.* Precious‐metal‐free electrocatalysts for activation of hydrogen evolution with nonmetallic electron donor: chemical composition controllable phosphorous doped vanadium carbide MXene. *Advanced Functional Materials* **29**, 1903443, doi:10.1002/adfm.201903443 (2019).

39	Handoko, A. D. *et al.* Tuning the basal plane functionalization of two-dimensional metal carbides (MXenes) to control hydrogen evolution activity. *ACS Applied Energy Materials* **1**, 173-180, doi:10.1021/acsaem.7b00054 (2018).

40	Intikhab, S. *et al.* Stoichiometry and surface structure dependence of hydrogen evolution reaction activity and stability of Mo_x_C MXenes. *Journal of Catalysis* **371**, 325-332, doi:<https://doi.org/10.1016/j.jcat.2019.01.037> (2019).

41	Meshkian, R. *et al.* W-based atomic laminates and their 2D derivative W_1.33_C MXene with vacancy ordering. *Advanced Materials* **30**, 1706409, doi:10.1002/adma.201706409 (2018).
